# How to predict effective drug combinations – moving beyond synergy scores

**DOI:** 10.1016/j.isci.2025.112622

**Published:** 2025-05-09

**Authors:** Lea Eckhart, Kerstin Lenhof, Lutz Herrmann, Lisa-Marie Rolli, Hans-Peter Lenhof

**Affiliations:** 1 Center for Bioinformatics, Saarland Informatics Campus, Saarland University, Saarbrücken, 66123 Saarland, Germany; 2 Computational Biology Group, Department of Biosystems Science and Engineering, ETH Zürich, Basel, 4056 Basel-Stadt, Switzerland

**Keywords:** Machine learning, Pharmacy

## Abstract

To improve our understanding of multi-drug therapies, cancer cell line panels screened with drug combinations are frequently studied using machine learning (ML). ML models trained on such data typically focus on predicting synergy scores that support drug development and repurposing efforts but have limitations when deriving personalized treatment recommendations. To simulate a more realistic personalized treatment scenario, we pioneer ML models that make dose-specific predictions of the relative growth inhibition (instead of synergy scores), and that can be applied to previously unseen cell lines. Our approach is highly flexible: it enables the reconstruction of dose-response curves and matrices, as well as various measures of drug sensitivity (and synergy) from model predictions, which can finally even be used to derive cell line-specific prioritizations of both mono- and combination therapies.

## Highlights

• Synergy scores have limitations for personalized treatment recommendation
• We pioneer dose-specific predictions of multi-drug sensitivity for unseen cell lines
• Benchmarking: Random forests using MACCS fingerprints performed best
• We introduce a drug combination sensitivity measure enabling treatment prioritization

## Graphical abstract

**Figure:**
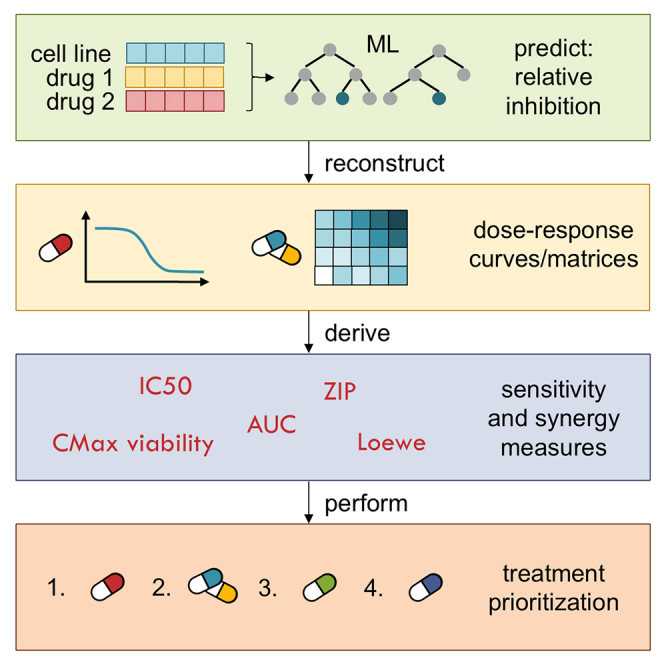

## Introduction

Tailoring drug treatments to the individual patient is a major goal of cancer research. Due to ethical concerns and limited availability of tumor material, relationships between molecular properties of cancer cells and their drug responses are generally not studied on humans directly, but instead using model systems, most prominently, cell lines. For monotherapy, large cell line panels such as the *Genomics of Drug Sensitivity in Cancer* (GDSC) database^1^^,^^2^ have been available for more than a decade, providing both molecular characterizations and drug screening data of cancer cell lines. However, combination therapies are frequently preferred over monotherapies for cancer treatment due to increased efficacy and a decreased risk of treatment resistance.^3^ More recently, large data resources have also become available for drug combination screens: In 2019, the DrugComb data portal was introduced,^4^ which to date accumulates harmonized results of drug screens for mono- and combination therapies from 37 different sources.^5^

Databases like the GDSC or DrugComb enable the systematic evaluation of the effect that different drugs have on different types of cancer cells. Thus, two main use cases that can be addressed using these data include (1) making personalized treatment recommendations for a given patient (cell line) and (2) finding promising drugs or drug combinations that should be further explored, e.g., for drug repurposing or the development of novel (combination) therapies. Due to the complexity and high dimensionality of the data, machine learning (ML) is commonly used to address these tasks.

ML models trained on monotherapy drug responses can be used for both use cases (1) and (2) since they directly predict measures of drug effectiveness, such as the IC50 or AUC value. In comparison, most methods using drug combination data predict so-called drug synergy scores.^6^^,^^7^ These scores quantify the synergistic or antagonistic potential of two compounds for a given cell line by comparing their combined effect on cell growth to the expected effect obtained from a baseline model that assumes no synergism or antagonism.^8^ Synergy scores are usually suited for the second task but less applicable for the first one, as discussed in detail in this manuscript. Mainly, a high synergy between two compounds does not guarantee that the respective combination treatment will be highly effective overall.^5^ It simply indicates that the combination treatment is more effective than what would be anticipated from the drugs’ monotherapy responses. Moreover, Palmer and Sorger found that the benefit of most combination therapies in clinical trials cannot be explained by synergy but rather by independent drug action.^9^ Another drawback of synergy scores is that they are typically aggregated over multiple drug concentrations that do not necessarily correspond well to clinically feasible concentration ranges.^10^

In this manuscript, we aim to address the task of combination drug response prediction by focusing on treatment sensitivity rather than synergy. To this end, we first conducted an extensive literature review of 55 state-of-the-art approaches for drug sensitivity and synergy prediction, which can be found in Table S1. Among the reviewed approaches, 32 are designed specifically for monotherapies and cannot directly be applied to data from combination treatments. Of the 23 approaches applicable to drug combination data, only 14 incorporate cell line features, e.g., gene expression profiles, in their input. This is crucial for making personalized treatment recommendations, where the drug response depends on the molecular properties of the individual cancer (here: cell line). The remaining approaches, with only two exceptions, focus on predicting synergy scores aggregated over multiple concentrations. These two exceptions are the models developed by Zheng et al.^5^ and comboFM by Julkunen et al.^11^ Both methods are designed to predict the concentration- and cell line–specific sensitivity of multi-drug treatments: Zheng et al. trained a CatBoost model that predicts the relative inhibition of two drugs at given concentrations for a given cell line. Similarly, comboFM employs higher-order factorization machines (HOFMs) to predict relative cell growth. Thus, both approaches can predict combination sensitivity. The strategy of predicting dose-specific drug responses enables the calculation of arbitrary sensitivity or synergy measures from the model predictions. The importance of developing models capable of making dose-specific predictions is also emphasized in Kong et al.’s systematic review on drug combination prediction.^12^

A limitation of the approaches by Zheng et al. and Julkunen et al. is that they are not applicable to make predictions for previously unseen cell lines (i.e., cell lines that were not included in the training data): They employ a one-hot encoding of cell lines in the model input such that the cell lines have to be known during training already. Consequently, these models are difficult to apply for personalized treatment recommendations, where predictions should be made for a previously unseen patient (cell line). According to Codice et al., this *cell-blind* setting is frequently overlooked or insufficiently evaluated in ML-based drug response prediction.^13^

Based on our literature review, there is currently no model for predicting drug combination sensitivity that can make dose-specific predictions for previously unseen cell lines. Thus, in this manuscript, we pioneer such models to mimic a personalized treatment scenario where the most effective treatment options for a given patient should be identified. Our contributions can be summarized as follows.

(1) We developed ML models to meet the demand for cell line–specific, dose-dependent predictions of drug combination sensitivity. Instead of predicting an aggregated measure of treatment response, our models predict the relative inhibition at arbitrary treatment concentrations provided in the model input.
(2) To determine how this novel prediction task can be modeled best, we systematically benchmarked different ML algorithms (neural networks, random forest, elastic net) and different drug representations in the model input (MACCS fingerprints, physico-chemical properties, drug targets). Our results show that random forests outperform the other algorithms in all investigated settings, while the drug representations were less decisive for performance. This evaluation can serve as a robust starting point for future model development.
(3) Our model architecture enables reconstructing various drug sensitivity or synergy measures from the predictions, including dose-response curves and matrices, as well as IC50 values or synergy scores. This versatility makes our models suited for a broad range of applications.
(4) For monotherapies, we reconstruct IC50 values and our recently proposed sensitivity measure called CMax viability.^14^ For combination therapies, we extend the CMax viability to be applicable to drug combinations. Our measure overcomes the limitations of existing sensitivity measures and is comparable across drugs and drug combinations. Furthermore, our analyses highlight a widely spread issue of evaluating the performance of multi-drug models.
(5) By integrating our models with the CMax viability, we can prioritize mono- and combination therapies. Drug prioritization, i.e., ranking drugs by their predicted effectiveness for a given cell line (patient), is a major goal in personalized medicine: it exceeds the mere prediction of sensitivity measures and moves toward deriving actual treatment recommendations.

## Results

### Challenges of synergy scores for recommending personalized treatments

The idea behind synergy scores is to measure the synergistic or antagonistic potential of two compounds for a given cell line by comparing their experimentally measured combined effect on cell survival to the expected effect obtained from a baseline model that assumes no synergism or antagonism.^8^ The baseline model is derived from monotherapy data of both compounds. It estimates the combined effect of the two compounds at the concentrations that were tested in the actual combination screening. The baseline and actually measured treatment responses are then subtracted from each other and the result is averaged over all concentration combinations to obtain a final synergy score.^10^ Prominent examples of synergy scores that differ solely in their computation of the baseline are the Loewe,^15^ Bliss,^16^ HSA,^17^ and ZIP^8^ scores. For each of these scores, values >0 indicate synergism and values <0 indicate antagonism. A detailed description of the scores can be found in the Supplement (Methods S1).

Undoubtedly, estimating the synergistic potential of compound combinations through synergy scores can be valuable for identifying promising combination treatments to undergo more detailed screening, the development of novel compounds, or drug repurposing. However, there are known limitations of synergy scores, which have been summarized and extensively discussed in a review by Vlot et al.^10^ They also investigated the agreement and across-batch reproducibility of four synergy scores (Loewe, HSA, ZIP, and Bliss) using a large-scale drug combination dataset. Their findings can be briefly summarized as follows: First, each synergy score is based on a set of model assumptions that differ between scores and may also be violated by real-world data.^18^^,^^19^ These varying model assumptions might explain the moderate to low correlation observed by Vlot et al. between the different scores calculated on the same data. Furthermore, while complete disagreement (synergism vs. antagonism) between scores was rare, Vlot et al. identified several scenarios where scores are likely to disagree, which could typically be retraced to a violation of model assumptions. Interestingly, although Vlot et al. report a strong correlation between the measured drug responses in terms of viability, the derived synergy scores are comparatively difficult to reproduce in replicated experiments.

Based on these findings, Vlot et al. advocate against the automated analysis of large-scale data using individual synergy scores. Instead, they recommend a careful investigation of the respective dose-response curves to then select an appropriate score.

We agree with these conclusions by Vlot et al. and would like to emphasize further points that make synergy scores difficult to use and interpret, especially for personalized treatment recommendations: A methodological criticism of synergy scores is that they are an aggregated measure over concentration ranges. The choice of meaningful concentration ranges is especially challenging for experimental drugs but crucial to draw meaningful conclusions for personalized medicine. For monotherapies, we have previously shown that the screened concentration ranges in the GDSC database do not correspond well to clinically feasible treatment concentrations.^20^ For combination therapies, similar observations can be made for the DrugComb database (cf. Figure S1, where we compare the screened concentrations to clinically feasible treatment concentrations).

Another major factor that hampers the use of synergy scores for treatment recommendation is that a high synergy between two compounds solely implies that the combination treatment is more effective than expected from the monotherapy responses of these two compounds. However, it does not guarantee an overall high effectiveness in terms of large relative inhibition of the combination treatment.^5^ To substantiate this theoretical argument, we investigated the correlation between the synergy scores and inhibition values provided by DrugComb. More precisely, we computed the Pearson correlation coefficient (PCC) between the synergy score and maximal inhibition obtained by each experiment (i.e., one cell line being treated with one drug combination). Correlations were low for all synergy scores (ZIP: 0.06, Loewe: 0.02, Bliss: 0.06) other than HSA (0.43), showing that a high synergy does not imply a high inhibition. Furthermore, we investigated the maximal inhibition of the 100 most synergistic experiments of each score. The results are provided in Figure S2 and show that, even among the most synergistic experiments, small or even negative inhibitions frequently occur, especially for the Bliss and ZIP scores.

Likewise, in a clinical setting, combination synergy is not the most conclusive factor for treatment success: Palmer and Sorger found that the benefit of most combination therapies in clinical trials can be explained by independent drug action rather than synergy.^9^ This underscores that synergy scores lack expressiveness for deriving personalized treatment recommendations.

### Moving beyond synergy scores

Given the limitations of synergy scores, particularly in the context of treatment recommendations, we focus on sensitivity prediction instead of synergy prediction. While there are numerous methods for predicting synergy,^6^^,^^7^ sensitivity prediction of drug combinations is relatively underexplored, especially when the goal is to make predictions for previously unseen cell lines, as outlined in the Introduction section (cf. related work in Table S1).

Treatment sensitivity is typically quantified using measures like the IC50 value and AUC for monotherapies and the CSS^21^ for combination therapies. However, these measures have limitations that impede their suitability in deriving personalized treatment recommendations. First, most sensitivity measures depend strongly on the investigated concentration ranges. For example, the AUC is calculated by integrating the dose-response curve over the investigated concentration range. Similarly, the CSS is based on drug-specific AUC values. Thus, poorly chosen concentration ranges can over- or underestimate sensitivity. Second, commonly used sensitivity measures are not comparable across compounds.^14^ Consequently, they cannot be applied to directly compare the effectiveness of different treatment options for a specific patient (cell line).

To overcome these challenges, we recently developed a sensitivity measure called the *CMax viability* (cf. STAR Methods for details)^14^: For monotherapies, the CMax viability of a cell line for a drug is defined as the relative viability after treatment with the drug’s CMax concentration (i.e., the peak plasma concentration after administering the highest clinically recommended dose^22^). Thus, the CMax viability estimates the maximal effect a treatment can realistically achieve. It ranges from 0 to 1, and smaller values indicate a higher treatment effectiveness.

Our measure was initially designed for monotherapies. In this manuscript, we introduce an extension for two-drug combinations described in the STAR Methods. Briefly, the *combination CMax viability* estimates the effect of a combination therapy when both drugs are administered at concentrations that do not exceed their respective CMax. Unlike conventional sensitivity measures, the CMax viability is comparable across drugs^14^ and drug combinations. Consequently, it can be used to prioritize drugs and combinations for a given cell line (i.e., rank them by their effectiveness, cf. Section treatment prioritization).

Certainly, the CMax viability is a valuable indicator of the maximum effect a treatment can achieve. However, there may be narrow concentration windows with beneficial or even synergistic effects, especially for drug combinations. Moreover, there may be patient-specific dosing requirements regarding, e.g., age, weight, or concurrent medications. Thus, we propose to go beyond the mere prediction of CMax viabilities. Instead, we advocate for and implement models predicting dose-specific drug sensitivity, which is also recommended in a recent review by Kong et al.^12^ Our models can make predictions for arbitrary concentrations specified in the input. Thus, sensitivity can be estimated at treatment concentrations relevant to the individual. Moreover, entire dose-response curves and matrices can be derived from the predictions. From these curves/matrices, all standard measures of sensitivity (or synergy) can be obtained. Thus, our approach is highly flexible and applicable not only for personalized treatment recommendations but also for identifying promising drug combinations to undergo *in vitro* screening or for drug repurposing. Moreover, by investigating the underlying curves/matrices, we can ensure that assumptions are met before calculating sensitivity or synergy measures.

### Predicting relative inhibitions

In the following, we analyze how accurately drug responses from the DrugComb database can be predicted for both mono- and combination therapies. More precisely, given (1) a cancer cell line of interest, (2) one or two treatment drugs, and (3) the corresponding drug concentration(s), our models predict the relative inhibition. The relative inhibition quantifies how much a drug treatment inhibits a cell line’s growth compared to an untreated control. In our analyses, relative inhibitions are in range [−200,200]. Values >0 indicate that the treatment inhibited growth and values <0 indicate an increase in growth (see STAR Methods for details).

We compare different ML algorithms and model inputs and investigate the reconstruction of sensitivity measures from the model predictions. Additionally, we show how both mono- and combination therapies can be ranked by their effectiveness for a given cell line using our recently developed sensitivity measure, the CMax viability.^14^

#### Model design

**Figure 1:**
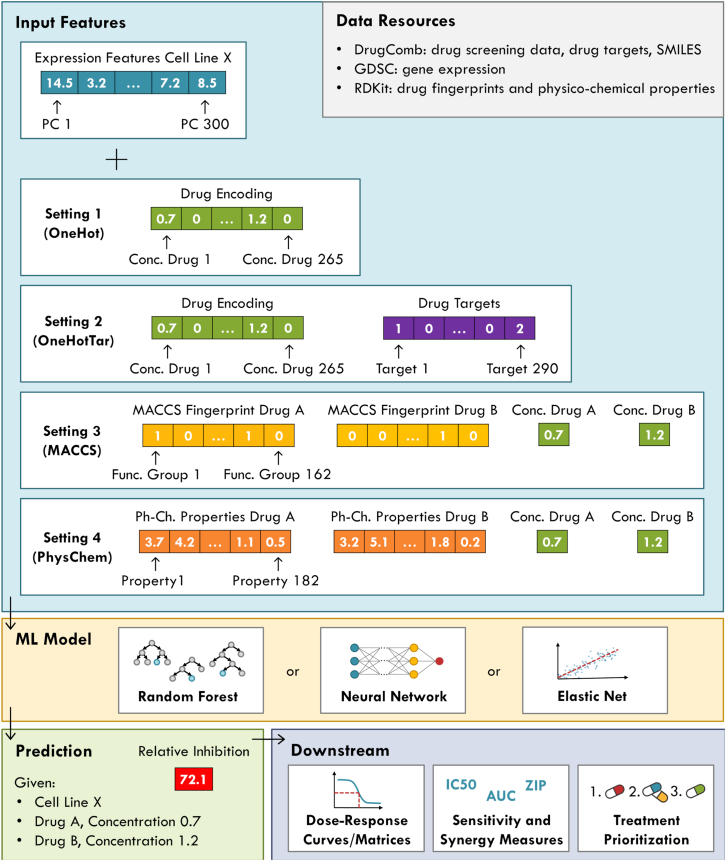
Prediction pipeline This figure summarizes our pipeline for the prediction of relative inhibitions. The large blue box depicts the different types of input features and representations we investigated. The gray box at the top right lists our data resources. The yellow box shows the different ML algorithms we used. The green box at the bottom depicts the model output, i.e., the relative inhibition for a given cell-drug-drug combination at defined treatment concentrations. Lastly, the purple box shows potential downstream analyses that can be performed based on the model predictions.

We trained multi-drug models that predict the relative inhibition for a given cell line being treated with given concentrations of one or two drug(s). The model inputs comprise cell line features derived from a principal component analysis (PCA) of gene expression values (see STAR Methods for details), a representation of the applied drugs, and the corresponding drug concentrations. For the representation of drugs, we investigated four different settings, which are depicted in Figure 1 (see STAR Methods for details).

##### Setting 1 (OneHot)

In this setting, drugs in the model input are represented through a 265-dimensional encoding where each feature corresponds to one of the 265 drugs in our dataset (cf. STAR Methods). If a drug is part of the current sample (i.e., the currently considered combination of a cell line, treatment drug(s), and the respective drug concentration(s)), its feature is set to the corresponding treatment concentration, otherwise it is set to 0.

##### Setting 2 (OneHotTar)

This setting uses the same concentration encoding as Setting 1 but additionally includes 290 features representing drug target molecules. Each feature is set to the number of drugs in the current sample that target the corresponding molecule.

##### Setting 3 (MACCS)

In this setting, each input drug is represented by a 162-dimensional binary *molecular access system* (MACCS) fingerprint.^23^ Additionally, one input feature for each drug is needed to denote its treatment concentration. To encode monotherapies, one of the fingerprints and the corresponding concentration are set to 0.

##### Setting 4 (PhysChem)

This setting is similar to Setting 3 but replaces the MACCS fingerprint with 182 numerical physico-chemical descriptors that denote different properties of the respective drugs, such as the molecular weight, number of valence electrons, or the logP value that measures lipophilicity.

Depending on the desired application, the different settings provide different benefits: Settings 3 and 4 allow making predictions for arbitrary drug molecules, given that their MACCS fingerprint or physico-chemical properties are known. Consequently, the resulting models can be used to make predictions for previously unseen compounds, e.g., newly developed ones. In contrast, models derived from Setting 1 and 2 are limited to those 265 drugs that were present in our dataset and hence encoded in the input. However, these models can not only make predictions for single drugs and two-drug combinations but even for treatments using three or more drugs simultaneously. While three-drug combination therapies have already been approved for cancer treatment by the United States Food and Drug Administration (FDA),^24^ DrugComb does not provide such data.

We used three different ML algorithms for model training, namely neural networks, random forests, and elastic nets. Notably, the cell lines contained in the training and test sets are disjoint (cf. STAR Methods). Thereby, the test data mimic the scenario of making predictions for a previously unseen patient.

### Overall performance comparison

**Figure 2:**
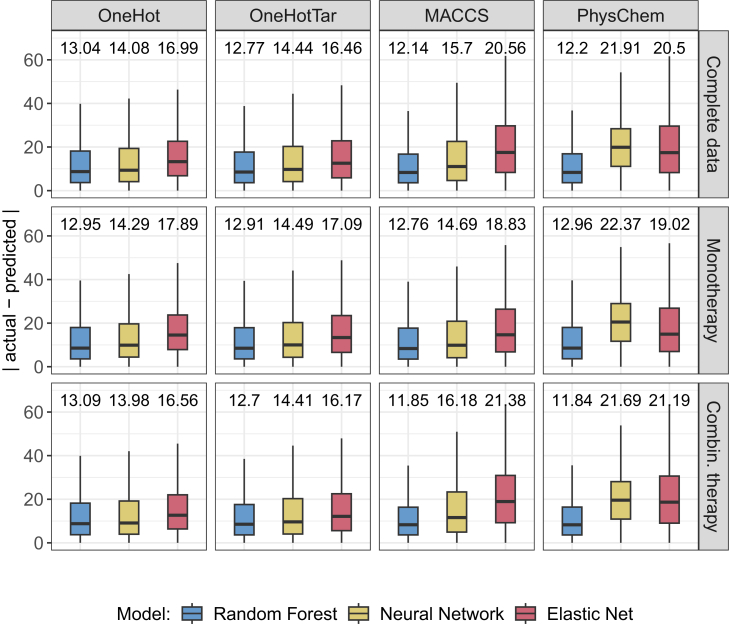
Test set performance This figure shows the prediction errors (in terms of the absolute difference between actual and predicted values) for each setting (columns) and each investigated ML algorithm (coloring). The first row shows the results for the entire test dataset, while the second and third row show the results for the data subsets corresponding to mono- and combination therapies, respectively. Data are represented as boxplots where the box denotes the interquartile range between the first quartile (25th percentile) and third quartile (75th percentile) of the data. The black horizontal line inside each box denotes the median, and the whiskers extend to the largest/smallest values within 1.5 times the interquartile range. Outliers are not shown. On top of each boxplot, the mean absolute error (MAE) is shown. See Figures S3–S5 for statistical evaluation and Figures S6 and S7 for Pearson correlation coefficients and $R^{2}$ values.

In our first analysis, we investigated which combination of the three investigated ML algorithms and four investigated drug representations can predict relative inhibitions most accurately. Figure 2 shows the performance of all investigated models in terms of test mean absolute error (MAE). The first row depicts the results for the entire test data, while the second and third row focus on the data subsets representing mono- and combination therapies, respectively. Across all four settings, random forests resulted in the lowest error, followed by neural networks, while elastic net had the worst performance. An exception is the PhysChem setting, where neural networks were outperformed by elastic net.

The overall smallest test error (MAE 12.14) was achieved using a random forest with MACCS fingerprints as input. Additionally, even the worst performing random forest model (OneHot, MAE of 13.04) still outperforms the best neural network (OneHot, MAE 14.08) and elastic net (OneHotTar, MAE of 16.46) models. Thus, the choice of ML algorithm seems to have a stronger impact on performance than the choice of input features, even though the different input representations differ considerably. Notably, the addition of drug targets slightly improves predictions for random forest and elastic net but has the opposite effect for neural networks. In Figures S3–S5, we provide a statistical evaluation of performance differences between the models (cf. STAR Methods). Differences were significant for almost all pairwise comparisons. Effect sizes range from 0 to 0.59 (mean: 0.29) and mirror the general trends described above.

To further contextualize the obtained errors, we compare them to two baseline models: A simple baseline model that always predicts the mean of the training data has a test MAE of 24.2. A more advanced baseline that always predicts the mean inhibition per drug for monotherapies and the mean inhibition of the combination for combination therapies has a test MAE of 19.74. Consequently, our best model (MACCS random forest) improves these baselines by 50% and 37%, respectively. While all of the random forest models outperform the baseline, some elastic nets and neural networks are not superior to the baselines.

When investigating mono- and combination therapies separately (cf. row 2 and 3 of Figure 2), the same overall trends can be observed, with the random forest model with MACCS features again having the smallest error. Generally, both types of therapies can be predicted similarly well, even though the training data contains slightly more combination (60%) than monotherapy data (40%).

Besides the MAE, we also investigated the PCC between the actual and predicted inhibitions. The overall PCC for the MACCS random forest was 0.8 (0.77 and 0.82 for mono- and combination therapies, respectively). However, a problem that is frequently overlooked^13^^,^^25^ is that computing correlations across the entire data artificially increases the PCC: since some drugs/combinations generally have lower/higher inhibitions than others, even mean predictions for each drug/combination (requiring no ML at all) would result in a correlation above 0.^13^ Thus, we computed the mean per-drug PCC for monotherapies (0.58) and the mean per-combination PCC for combination therapies (0.56) (see also Figure S6 for boxplots). These values have a similar magnitude to what we previously observed for monotherapy sensitivity prediction.^14^ PCC and $R^{2}$ values for each model are provided in Figure S7.

Note that Zheng et al.^5^ and Julkunen et al.^11^ also provide overall correlations and errors for the prediction of relative inhibition/growth (cf. Introduction). However, their results are not comparable to ours since we investigate the performance for unknown cell lines, which cannot be evaluated using the other two methods. It is known that the cell-line blind scenario increases errors considerably compared to making predictions for known cell lines.^26^^,^^27^ To, nevertheless, assess how our random forest MACCS model would perform for known cell lines, we retrained the model using a random split of the available data into a training (80%) and test set (20%). This split does not guarantee that cell lines in the test set were unseen during model training. Note that we still ensured that duplicated entries denoting the same treatment are either exclusively contained in the training or the test set (cf. STAR Methods). With a PCC of 0.96 and RMSE (root mean squared error) of 8.41, our performance for known cell lines is comparable to that reported by Zheng et al. (PCC = 0.98, RMSE = 7.12)^5^ and Julkunen et al. (PCC = 0.97, RMSE = 9.86 in cross-validation; PCC = 0.92 on validation data).^11^ However, the dataset used in our analyses is much larger and more heterogeneous comprising 947 cell lines, 265 drugs, and 9,535 drug combinations from different sources. In contrast, Zheng et al. employed solely the O’Neil dataset (39 cell lines, 38 drugs, 583 drug combinations),^28^ which is known to be of high quality,^4^^,^^5^ whereas Julkunen et al. employed solely the AstraZeneca DREAM dataset (85 cell lines, 118 drugs, 910 drug combinations).^6^

### Range performance comparison

Next, we investigated whether certain inhibition ranges can be predicted more accurately than others. We were motivated to perform this analysis by the observation that the under-representation of certain response intervals in the training data can negatively affect their prediction quality.^29^ In particular, data points with high inhibition are commonly underrepresented in drug screening datasets,^30^^,^^31^^,^^32^ which can negatively affect their prediction. They are, however, of particular interest for personalized therapy since they represent cases where the drug treatment greatly reduced the number of viable cells, i.e., cases of effective treatment.

**Figure 3:**
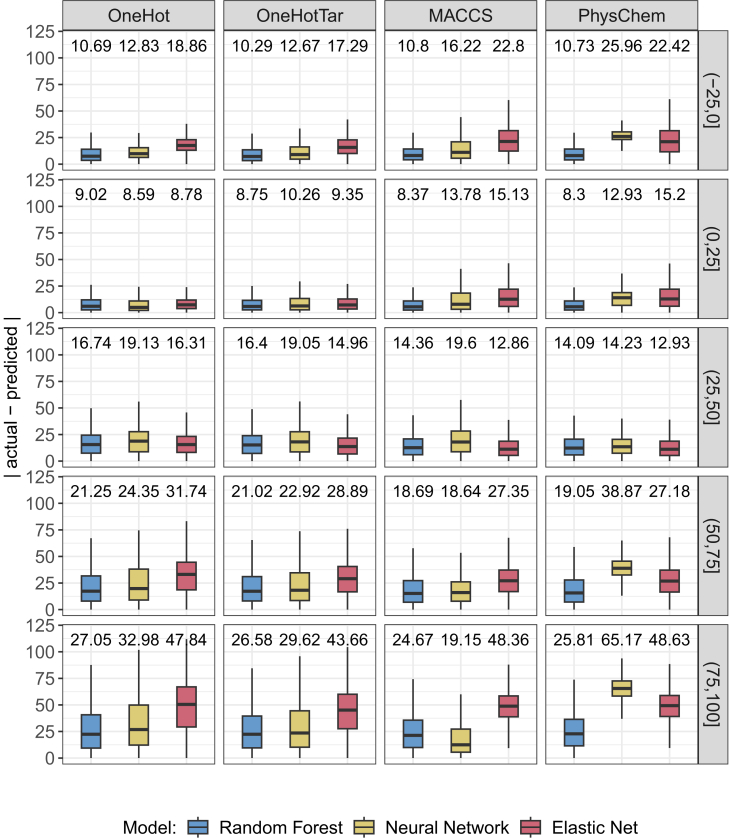
Test set performance for different inhibition ranges This figure shows the prediction errors (in terms of the absolute difference between actual and predicted values) for each setting (columns) and each investigated ML algorithm (coloring). Each row shows the performance for a different interval of actual relative inhibitions. Data are represented as boxplots where the box denotes the interquartile range between the first quartile (25th percentile) and third quartile (75th percentile) of the data. The black horizontal line inside each box denotes the median, and the whiskers extend to the largest/smallest values within 1.5 times the interquartile range. Outliers are not shown. On top of each boxplot, the mean absolute error (MAE) is shown. See also Figures S8–S17 for statistical evaluations, PCC, and $R^{2}$ values.

For this analysis, we again investigated all three ML algorithms and four input representations. Figure 3 shows the distribution of test MAEs for different inhibition intervals in range (−25,100]. This range covers 99% of the training and test data. Predictions are on average most accurate in the interval (0,25] followed by the interval (−25,0]. As the actual inhibition increases, the error increases as well (cf. Figures S8–S17 for statistical evaluations, PCC, and $R^{2}$ values). In line with our motivation above, this observation could be explained by the amount of available training data for each interval: Most data are located in the intervals (0,25] (41%) and (−25,0] (25%), while each of the other intervals is only covered by around 10% of the data. Thus, highly effective treatments are under-represented.

To counteract this under-representation for monotherapies, we developed SAURON-RF, a random forest-based model that is designed to improve predictions of drug-sensitive samples for both classification and regression.^14^^,^^29^ To this end, SAURON-RF relies (among other things) on sample-specific weights to increase the importance of the under-represented intervals. Consequently, we also tried to incorporate sample weights into our models presented here. Unfortunately, the sample weights had only little impact on predictions, especially for the cases with highest inhibition (see Figure S18 for a performance comparison of the MACCS random forest model with and without sample weights).

### Correlation of duplicated entries

For the models using one-hot encodings (settings OneHot and OneHotTar), each drug has a designated input node. This is not the case for the MACCS and PhysChem settings, where the same combination treatment can be described by two different input representations through swapping the order of the considered drugs in the input vector (cf. input visualization in Figure 1 and STAR Methods). However, the model output should not depend on the order of the drugs in the input, i.e., it should not depend on whether the drug features and concentration of a drug A are located in front of or behind those of a drug B in the input vector. Hence, we decided to include both input representations into the training and test data of our models.

**Figure 4:**
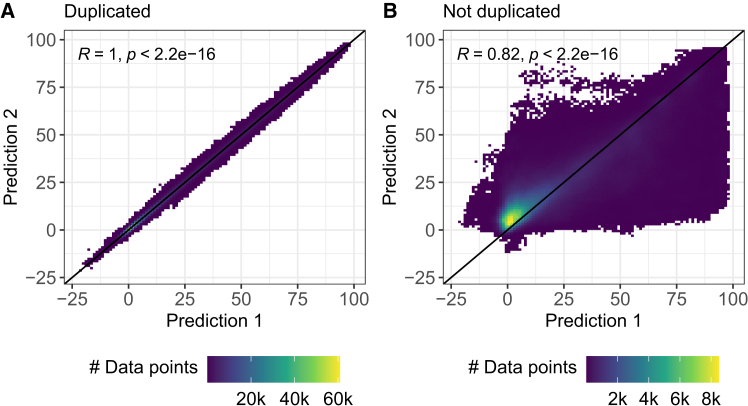
Correlation of duplicated entries from the test data This figure shows the correlation between the model predictions for duplicated entries using the random forest MACCS model. Duplicated entries refer to the same drug-drug-cell combination and the same treatment concentrations but can be represented by two different model inputs through swapping the features of the respective drugs (cf. STAR Methods and Figure 1) (A) shows the test predictions when including duplicated entries into the training data, while (B) shows the predictions when training only on non-duplicated entries. In both figures, the black diagonal line represents the identity, R denotes the Pearson correlation between the predictions, and *p* is the corresponding *p*-value of a two-sided t test for R. See Figure S5 for results of the random forest PhysChem model.

Ideally, predictions for both input representations should correlate well. Figure 4A shows the correlation of predictions for the best-performing random forest model trained using MACCS fingerprints. As desired, both predictions are highly correlated (PCC ≈ 1) and the mean absolute difference between them is very small (0.8). Figure 4B shows the same analysis for a model where we removed the duplicated entries from the training data. Even though the correlation is still high (PCC = 0.82), it decreased strongly, while prediction differences increased notably to 9.12 on average. The mean PCCs per drug (for monotherapies) and per drug combination are 0.98 and 0.97 for the duplicated training data and decrease to 0.78 and 0.86 for the non-duplicated training data, respectively. This is also represented in the test error where the model with duplicated training entries achieved an MAE of 12.39 compared to 14.6 for non-duplicated entries. Similar trends can also be observed for the PhysChem setting (see Figure S5). Based on these findings, we conclude that it is advisable to train models using all possible input representations of a treatment.

### Reconstruction of sensitivity measures

A benefit of predicting concentration-specific inhibition values is that based on the model’s predictions, dose-response curves (and matrices) can be reconstructed. These can in turn be used to compute various measures of drug sensitivity or synergy.

We first investigated how accurately dose-response curves can be reconstructed from the predictions of the MACCS random forest. To this end, we used the actual and predicted inhibition values to fit dose-response curves for each cell line and drug combination (cf. STAR Methods). Next, we computed the RMSE between the actual and predicted curves. This resulted in a mean RMSE of 0.13. For reference, the GDSC database considers curves with RMSE < 0.3 to be of satisfactory quality.^33^^,^^34^ In fact, 93% of the reconstructed curves have an RMSE < 0.3, indicating an accurate reconstruction in a majority of cases.

Since the focus of this paper is on sensitivity prediction and Vlot et al. discourage the computation of arbitrary synergy scores on large-scale data,^10^ we reconstructed three measures of drug sensitivity, namely IC50 and CMax viability values for monotherapies, and a modification of the CMax viability for drug combinations, which we call the *combination CMax viability* (cf. STAR Methods for details). Here, we focus mainly on evaluating the reconstruction of the CMax viability. Analogous results for IC50 values can be found in Figure S21.

**Figure 5:**
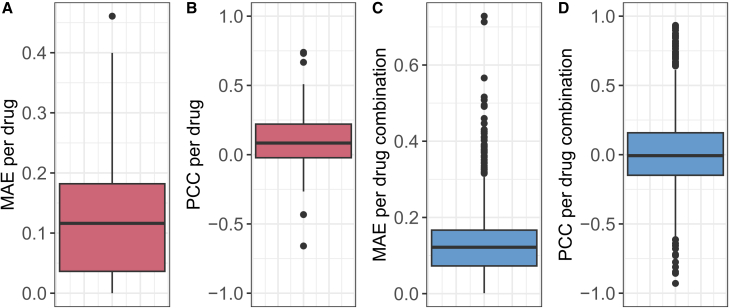
Reconstruction of (combination) CMax viabilities from predicted dose-response curves/matrices (A) and (B) (red) show the distribution of MAE and PCC per drug for the reconstruction of CMax viabilities using dose-response curves fit on the test set monotherapy data. (C) and (D) (blue) show the distribution of MAE and PCC per dug combination for the reconstruction of combination CMax viabilities using dose-response matrices derived from the test set drug combination data. Data are represented as boxplots where the box denotes the interquartile range between the first quartile (25th percentile) and third quartile (75th percentile) of the data. The black horizontal line inside each box denotes the median, and the whiskers extend to the largest/smallest values within 1.5 times the interquartile range. See also Figures S21 and S22.

In total, we were able to compute both the actual and predicted monotherapy CMax viabilities for 7,352 out of 32,564 cell line-drug combinations. The decreased number of combinations stems from the fact that CMax concentrations were only available for 77 of the investigated drugs. Figure 5A depicts the prediction errors for the reconstructed monotherapy CMax viability. The mean MAE averaged over all drugs is 0.12 and the mean MSE is 0.04. The results are comparable to the error we previously achieved when predicting CMax viabilities directly using our recently published algorithm SAURON-RF^14^^,^^29^ (MSE = 0.03) or a slightly adjusted version of DeepDR by Chiu et al.^35^ (MSE = 0.09).^14^

A baseline error can be obtained from a model that, for every treatment concentration, predicts the mean inhibition for each drug obtained from the training data. For such a model, the CMax viability would also be predicted as this mean. This results in a baseline MAE of 0.2, which our model improves by 40%.

The overall PCC is 0.58 for the CMax viabilities and 0.41 for the baseline. However, the drug-specific PCC is only 0.1 (cf. Figure 5B). While a drug-specific baseline PCC cannot be computed for constant predictions, adding random noise with mean 0 to these constant predictions results in a baseline PCC of 0. Thus, our predictions improve this baseline but only slightly. When using our models to reconstruct IC50 values, we observe a similar phenomenon (overall PCC = 0.71, mean PCC per drug = 0.01, cf. Figure S21).

To investigate the reasons for these low drug-specific correlations, we developed and evaluated different hypotheses.

**Hypothesis 1**: The reconstructed dose-response curves might not accurately model the CMax viability in cases where concentrations exceeding the CMax concentration of the respective drug have not been screened.

**Evaluation 1:** Concentrations exceeding the CMax concentration were only screened for 63 of the 77 drugs, corresponding to 70% of the drug-cell line combinations in the test set. Thus, we evaluated the PCC only on those drug-cell line combinations. This increased the average PCC slightly from 0.1 to 0.12.

**Hypothesis 2**: Even if sufficiently large concentrations were screened, the increased prediction errors for data points with high inhibition (cf. Figure 3) might make the curve-fitting unreliable in areas of high inhibition, affecting the derived measures.

**Evaluation 2:** The IC50 value is designed to measure the drug response at a relative inhibition of 50%. To assess the performance at smaller inhibitions (i.e., higher viabilities), we reconstructed IC75 and IC90 values from the fitted curves. The IC75 (IC90) measures the drug concentration where a relative inhibition of 100%−75%=25% (100%−90%=10%) is reached. The average per-drug PCCs for the IC75 and IC90 reconstruction are 0.11 and 0.1, respectively, thus, there is no (strong) improvement compared to the IC50 predictions (cf. Figure S21).

**Hypothesis 3**: The two-step process of first reconstructing a curve and then deriving the CMax viability from the curve is inferior to directly predicting the CMax viability with our models.

**Evaluation 3:** Instead of deriving the CMax viability from the estimated dose-response curves, we used our model directly to predict the relative inhibition at the CMax concentration and converted this prediction into a relative viability. However, this did also not improve correlations (PCC 0.04, cf. Figure S22). Instead, the curve fitting seems to enhance predictions slightly (cf. also Figure S20), which is in line with the findings by Rahman and Pal.^36^

Unfortunately, none of these hypotheses provide a comprehensive explanation for the low PCCs. Thus, we conclude that even though prediction errors (MAE) are relatively small and comparable to our previous work, the derived measures only indicate a general trend in sensitivity but cannot be used to compare the effect of a drug monotherapy on different cell lines. For the combination CMax viability (26,946 drug-drug-cell line combinations), we obtained similar results that are depicted in Figures 5C and 5D.

Nevertheless, we would like to highlight that such an evaluation of drug-specific correlations as conducted here is frequently not performed for drug sensitivity and synergy prediction (cf. related work in Table S1). Thus, similar problems may often go undetected. In particular, neither Zheng et al.^5^ nor Julkunen et al.^11^ provide drug-/combination-specific correlations. For cell-blind evaluations on monotherapy data, we found three related approaches that provide drug-specific correlations: Our recently published method SAURON-RF achieves a mean PCC of 0.56 when directly predicting CMax viabilities using drug-specific models.^14^ In the same manuscript we also show that an adjusted version of the multi-drug model DeepDR by Chiu et al.^35^ achieves a PCC of 0 for the same task. In comparison, Chawla et al. employ multi-drug models for the prediction of IC50 values and achieve mean PCCs between ca. 0.18 and 0.5 for different ML algorithms. Lastly, Rahman and Pal achieve mean PCCs between 0.29 and 0.44 when reconstructing AUC values from predicted dose-response curves. While not directly comparable to our approach, these works underline that at least weak to moderate drug-specific correlations can be achieved: (1) when predicting CMax viabilities using models directly trained for this task, (2) when using multi-drug models, or (3) when deriving sensitivity measures from predicted curves. Yet, it remains to be investigated further if and how comparable results can be achieved when combining all three factors and also considering combination therapies, thereby enabling predictions for arbitrary drugs/combinations and measures, which we aim to achieve here.

### Treatment prioritization

**Figure 6:**
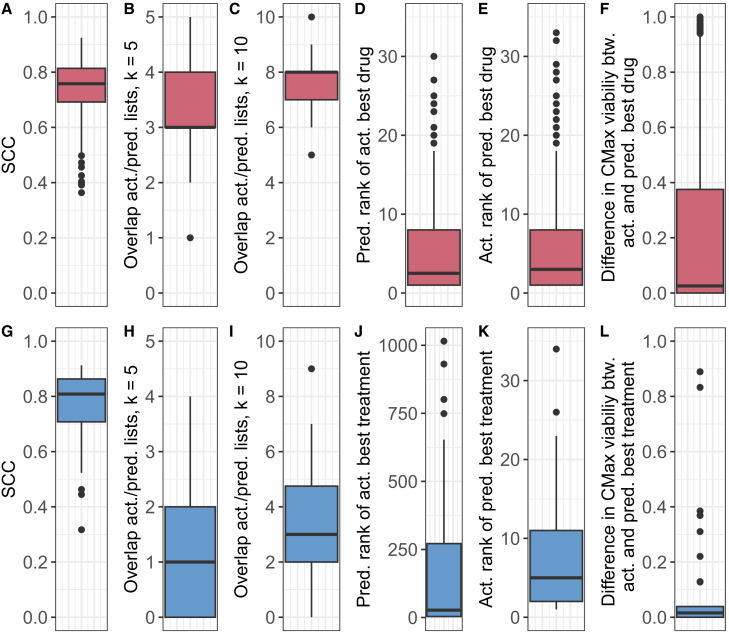
Treatment prioritization This figure depicts the test set prioritization results for mono- and combination therapies. Sub-figures A to F (red) focus on the prioritization of monotherapies including: (A) the SCC between the actual and predicted rankings for each cell line, (B)/(C) the intersection size between the 5/10 actual and predicted most effective treatments, (D) the predicted rank of the actual most effective treatment, (E) the actual rank of the treatment predicted to be most effective, and (F) the difference between the actual CMax viabilities for the actual and predicted most effective treatment. (G)–(L) (blue) show the analogous prioritization results when combining mono- and combination treatments into one list. Data are represented as boxplots where the box denotes the interquartile range between the first quartile (25th percentile) and third quartile (75th percentile) of the data. The black horizontal line inside each box denotes the median, and the whiskers extend to the largest/smallest values within 1.5 times the interquartile range. See also Figures S23 and S24 for further intersection sizes.

In our final analysis, we investigate how accurately drugs and drug combinations can be prioritized for a given cell line based on the MACCS random forest predictions: For each cell line in the test set, we used the computed CMax viabilities for the monotherapy and combination data to achieve a ranking of drugs and drug combinations from most to least effective (i.e., from smallest to largest CMax viability). Drug prioritization is supposed to mimic a personalized treatment scenario with the goal to achieve a list of most effective treatment suggestions for a given patient. The results are shown in Figure 6, where the first row shows the results for monotherapies only, while the second row shows the results when combining mono- and combination therapies into one list.

For monotherapies, the Spearman correlation coefficient (SCC) between the actual and predicted rankings was 0.74 (baseline (as defined in the previous section): 0.54). Our predictions clearly outperform the baseline. Still, the baseline correlation is relatively high, indicating that the differences in effectiveness between drugs are easier to predict than the differences between cell lines receiving the same treatment (as investigated in the previous section).

While an accurate ranking for the entire list is desirable, one would typically place more emphasis on the correct identification of the most effective treatments. Thus, we computed the mean overlap between the first *k* elements of the actual and predicted rankings. For monotherapies, the average length of the predicted drug lists is 31.15. The average overlap between the top k=5 and k=10 actual and predicted most effective drugs is 3.16 (baseline: 2.14) and 7.68 (baseline: 6.55), respectively. Results for further *k* are shown in Figure S23. Furthermore, the median rank of the actually most effective drug in the predicted ranking is 2.5 (baseline: 8), and the median rank of the drug predicted to be most effective in the actual list is 3 (baseline 6). The median difference between the true CMax viabilities of the actual most effective and predicted most effective drugs is only 0.02 (baseline 0.31). Thus, the predicted rankings recommend treatments that are similar in effectiveness to the actual best treatment.

The second row of Figure 6 shows the analogous prioritization results when combining mono- and combination treatments into one list. The SCC of 0.76 (baseline: 0.62) is comparable to the results for monotherapies. Since the average list length is much greater when including drug combinations (838.62), the overlaps at k=5 (1.26, baseline: 0.68) and k=10 (3.38, baseline: 2.09) are lower (cf. Figure S24 for further *k*. Furthermore, the median rank of the actually best treatment in the predicted list (27, baseline: 170.5) and of the predicted best treatment in the actual list (9.5, baseline: 12) decrease. Still, results clearly improve over the baseline. Furthermore, the median difference in viability between the actually most effective treatment and the treatment predicted to be most effective remains small (0.02, baseline 0.03).

## Discussion

Administering not only single but multiple drugs in combination is common in cancer treatment. However, while drug response datasets for monotherapy data have been available for more than a decade, large-scale datasets for combination therapy have only become publicly available more recently, e.g., through the DrugComb database.^4^^,^^5^ While the DrugComb data have extensively been studied for the prediction of drug synergy, they are still underused for the prediction of drug sensitivity, especially with the focus on making personalized treatment recommendations. For this application case, we found the scores that are widely used for synergy prediction less suited due to various reasons discussed in this manuscript. Thus, we argue that a more suitable approach for predicting drug combination sensitivity in a personalized treatment setting is the direct prediction of concentration-specific drug combination responses in the form of relative inhibitions (instead of synergy scores).

To show the capabilities of our approach in practice, we developed and evaluated several ML algorithms when applied to different types of input features and representations. Most notably, we evaluated our models in the often-neglected cell-blind scenario,^13^ thereby mimicking personalized drug predictions for a new patient. Our evaluations demonstrate that our models substantially improve baseline models and show very little variation when predicting the same treatment using different input representations. Apart from that, our models are also competitive with state-of-the-art approaches when making predictions for known cell lines. Furthermore, we reconstructed the *(combination) CMax viability*, a sensitivity measure that we recently developed for monotherapies,^14^ and that we extended to be amenable to combination drug response prediction in this manuscript. Using this measure, we were able to perform prioritization of both mono- and combination therapy options for unseen cell lines, which is the scenario that mimics personalized drug treatment recommendation most closely, where a sorted list of the most effective treatment options is desired.

### Limitations of the study

While our evaluations demonstrate the capabilities of our approach, this study also reveals weak points of the methodology that require attention during the development of future methods.

(1) We observed increased prediction errors for data samples with high inhibitions, i.e., cases of high treatment sensitivity, which are typically of particular interest for treatment recommendation. In monotherapy prediction, this issue is relatively well-known for classification but has rarely been discussed or addressed for regression.^29^^,^^32^ While we resolved this issue for monotherapy classification and regression in earlier work using sample-specific weights,^14^ such weights had little impact in the current study.
(2) Additionally, we noted that drug/combination-specific correlations between the reconstructed measures (IC50 and CMax viability) and the actual values derived from the dose response curves/matrices only barely surpass the baseline model. Interestingly, this issue is not uncommon: we recently observed similar tendencies for other multi-drug models performing monotherapy prediction.^14^ However, a comprehensive explanation for this observation remains elusive. Moreover, we found that this issue may usually go undetected since drug-specific measures are infrequently evaluated (cf. related work in Table S1).^13^ Thus, we encourage others to investigate and report drug/combination-specific correlations when developing novel prediction approaches.

Ovchinnikova et al. criticize that multi-drug models predicting measures like the IC50 or AUC frequently only learn to distinguish drugs (but not cell lines) since the value ranges vary between drugs.^25^ To overcome this issue, they propose normalizing the response measures per drug by using z-scores. However, for the following reasons we recommend exploring alternative strategies: (1) Both IC50 and AUC values are highly dependent on the tested concentration range, and z-score normalization does not solve this issue. (2) Z-scores assume that the data follows a normal distribution, which is generally not the case for drug response data. For example, the GDSC IC50 drug responses are typically bimodal.^30^ (3) By converting response measures into z-scores, information on the absolute effectiveness can be lost, which is undesirable for treatment recommendation. Thus, instead of using z-scores, we advocate performing a per-drug evaluation (as shown in this manuscript) to detect the issue. To resolve it, measures with across-drug comparability should be used to train models, e.g., the CMax viability. Additionally, since we observed that single-drug models do not suffer from this issue,^14^^,^^20^ we propose that it might be advisable to introduce drug-specific regularization terms into the training of multi-drug models.

Three main factors can be adjusted to potentially address such challenges, namely the choice of ML algorithm, the choice and representation of input features, and the used data.

#### ML algorithm

We investigated neural networks (highly popular for sensitivity and synergy prediction), random forests, and elastic nets. In our recently published benchmarking, we found both elastic nets and random forests outperform neural networks when predicting drug sensitivity.^20^ For the prediction of inhibitions, as investigated here, random forests are superior to the other algorithms. In general, a plethora of further potentially more sophisticated approaches can be used to model the prediction of inhibitions. For example, we could adjust the objective function optimized during model training to potentially improve the drug-specific predictions. However, as discussed in our benchmarking^20^ and also by Li et al.,^37^ more complex approaches are not necessarily superior to simpler ML algorithms, and careful evaluation is required to ensure a fair performance comparison.

#### Input features and representation

For the characterization of cell lines in the model input, several sources found gene expression to be the most informative omics-type for predicting drug responses.^35^^,^^38^^,^^39^ However, the inclusion of further omics or *a priori* knowledge, e.g., known sensitivity biomarkers or protein interactions, might improve predictions.

Similarly, further drug properties, e.g., Morgan fingerprints^40^ could be investigated, or graph neural networks could be employed to represent drugs as molecular graphs – both options might improve drug-specific predictions. However, the superiority of molecular graphs over conventional drug fingerprints for sensitivity/synergy prediction and drug discovery has been questioned.^41^^,^^42^

#### Dataset

With 947 cell lines, 265 drugs, and 9,535 drug combinations, the dataset investigated here is notably larger than those used by other approaches working on drug combination data.^5^^,^^11^^,^^21^^,^^26^^,^^43^^,^^44^^,^^45^ However, given the size of this dataset, hardware restrictions became a limiting factor for ML training. Despite training models on a compute cluster with machines of 500 gigabytes working memory, we had to reduce our data regarding the number of considered drugs and features (cf. STAR Methods).

Generally, a large amount of training data benefits model training and robustness. Yet, if the dataset is heterogeneous, e.g., due to different data sources as in DrugComb, this may decrease performance compared to models built and evaluated on a more homogeneous dataset. Even though Zagidullin et al. and Liu et al. found the reproducibility between replicates from different datasets in DrugComb satisfactory,^4^^,^^46^ disagreement between drug response data from different sources is a well-known problem.^39^^,^^47^^,^^48^ However, Vlot et al. found that, unlike synergy scores, relative viabilities (inhibitions) were highly correlated between different batches in their analysis of drug combination data.^10^ Similarly, we found only small variation in the inhibition values of replicates in our analyses (cf. STAR Methods). Especially for clinical applications, combining data from different sources (e.g., different hospitals) is essential, and models should be able to cope with some degree of heterogeneity. To this end, meta- or transfer-learning methods could also be leveraged.^49^ These methods might also be viable to translate the results of (bulk sequencing from) model systems to (single-cell) patient data to enable the use of prediction models in the clinic.^50^

Lastly, treatment efficacy is only one building block of treatment personalization in a clinical scenario. Treatment choices are also heavily impacted by potential side effects and toxicity that can significantly affect a patient’s well-being and may even be deadly. Estimating side effects is especially challenging when a patient is treated with multiple drugs at the same time. Zhao et al. recently published an extensive review on this topic, providing a plethora of resources for assessing side effects of single- and multi-drug treatments, including databases and ML-based prediction models.^51^

## Resource availability

### Lead contact

Requests for further information and resources should be directed to and will be fulfilled by the lead contact, Lea Eckhart (lea.eckhart@uni-saarland.de).

### Materials availability

This study did not generate new materials.

### Data and code availability

• We provide the SMILES, MACCS fingerprints and physico-chemical properties derived from RDKit,^52^ as well as the one-hot encoded target molecules of the investigated compounds at GitHub (https://github.com/unisb-bioinf/Drug_Combination_Sensitivity_Prediction). All other data reported in this paper will be shared by the lead contact upon request.
• Our code is also available at GitHub (https://github.com/unisb-bioinf/Drug_Combination_Sensitivity_Prediction).
• Any additional information required to reanalyze the data reported in this paper is available from the lead contact upon request.

## Acknowledgments

This work was supported by internal funds of 10.13039/501100005690Saarland University.

## Author contributions

L.E., K.L., and H.-P.L. conceived the study. L.E. designed the study. L.H. and L.E. collected and processed the data. L.-M.R. computed the drug properties. L.E. implemented the software and conducted the experiments. L.E. drafted the initial manuscript. L.E. and K.L. revised the manuscript. K.L. and H.-P.L. supervised the study. All authors analyzed the data, evaluated the results, and commented on the manuscript.

## Declaration of interests

The authors declare no competing interests.

## STAR★Methods

### Key resources table

**Table:** 

REAGENT or RESOURCE	SOURCE	IDENTIFIER
**Deposited data**		

Gene expression values of human cancer cell lines	Genomics of Drug Sensitivity in Cancer database	https://www.cancerrxgene.org/downloads/bulk_download
Drug response values of human cancer cell lines	DrugComb database	https://api.drugcomb.org/
Drug properties (SMILES drug representations, drug targets)	DrugComb database	https://api.drugcomb.org/
Drug properties (MACCS fingerprints, physico-chemical properties)	This paper	https://github.com/unisb-bioinf/Drug_Combination_Sensitivity_Prediction
CMax concentrations	Liston and Davis	https://doi.org/10.1158/1078-0432.ccr-16-3083

**Software and algorithms**		

Our prediction models	This paper	https://github.com/unisb-bioinf/Drug_Combination_Sensitivity_Prediction
Python 3.11	Python Software Foundation	https://www.python.org
RDKit 2023.3.2	Landrum et al.^52^	https://doi.org/10.5281/zenodo.8053810
scikit-learn 1.5.0	Pedregosa et al.^53^	https://scikit-learn.org/stable/index.html
tensorflow 2.16.1	Abadi et al.^54^	https://www.tensorflow.org/
R 3.6.3	R Foundation for Statistical Computing	https://www.r-project.org/
drc 3.0-1	Ritz et al.^55^	https://doi.org/10.32614/CRAN.package.drc
stats 3.6.3	R Foundation for Statistical Computing	https://www.r-project.org/
rcompanion 2.3.21	Rutgers Cooperative Extension	https://doi.org/10.32614/CRAN.package.rcompanion
pracma 2.4.2	Hans W. Borchers	https://doi.org/10.32614/CRAN.package.pracma

### Method details

#### Materials and data processing

##### Drug response data

Drug screening data for our analyses was obtained from the DrugComb database Version 1.5. More specifically, we employed the DrugComb API (https://api.drugcomb.org/) to download the list of all cell lines and their corresponding COSMIC IDs, the full list of drugs with their SMILE encodings and their target molecules, and the full dose-response matrices.

To assign the respective cell line and drug information to each dose-response matrix, we downloaded the core database from https://drugcomb.org/download, which provides a unique identifier for each dose-response experiment. Consequently, each database entry can be written as:

(cell_line,drug_row,drug_col,conc_row,conc_col,inhibition)

Here, cell_line is the COSMIC ID of the investigated cell line, and drug_row and drug_col are the names of the tested drugs. The entries conc_row and conc_col are the micromolar concentrations of the tested compounds. For monotherapies, one of the drug names is set to NULL and the corresponding concentration is set to 0. Finally, inhibition denotes the relative inhibition measured after administration of the denoted drug concentration(s) (see Data S1 for further information). Relative inhibitions >0 denote reduced cell growth through the drug treatment, while inhibitions <0 indicate increased growth.

We removed the following entries from the dataset:

• poor quality entries as defined by the authors of DrugComb^5^ with inhibition<−200 or inhibition>200
• entries where the concentration of all tested drugs is 0 (conc_row=conc_col=0)
• entries, where the corresponding cell line had no COSMIC ID or no gene expression data provided in the GDSC database

Additionally, we converted entries where drug_row and drug_col denote the same drug into monotherapies by summing the respective treatment concentrations and setting drug_col to NULL:

(cell_line,drug_row,NULL,conc_row+conc_col,0,inhibition)

Cases where two different drugs are provided but only one has a concentration >0 were modified to denote a monotherapy by replacing the drug with concentration 0 with NULL. Afterwards, all replicates involving the same cell line, the same drug(s), and same concentration(s) were averaged. Given the inhibition range of [−200,200], the average standard deviation between replicates was small (mean: 7, median 5). Lastly, we log1p-normalized (log1p(x)=log(x+1)) the concentration values in conc_row and conc_col.

To keep the dataset size manageable, we only considered entries involving those 265 drugs (cf. Table S2) for which at least 10,000 entries are provided after performing all the steps described above (cf. Discussion). Note that after this reduction still more than 10,000 entries remained for each of the drugs. In total, the final dataset consists of 5,291,424 entries covering 947 cell lines, 265 drugs, and 9,535 drug combinations.

Additionally, the CMax concentrations for 77 of the investigated drugs were obtained from Liston and Davis.^22^ The CMax value denotes the peak plasma concentration after administering the highest clinically recommended dose of a drug.^22^ In a recently published manuscript, we employed CMax to derive a novel drug sensitivity measure called the *CMax viability*, which will be described below.^14^ We also use this measure to perform drug prioritization in the results section.

##### Drug properties

For the representation of drugs in the inputs of our models, we investigated four different settings, which will be discussed below (cf. also our prediction pipeline in Figure 1). Using the SMILES drug representations provided by DrugComb, we used RDKit version 2023.3.2^52^ to calculate two types of drug features.

• binary MACCS fingerprints^23^ of length 166.
• 209 physico-chemical drug properties using the function CalcMolDescriptors from the rdkit.Chem.Descriptors module.^56^

We removed all properties that showed no variation across the investigated 265 drugs, resulting in MACCS fingerprints of length 162 and 182 physico-chemical properties.

Additionally, 735 drug target molecules for the investigated drugs were obtained from DrugComb.

##### Gene expression data

Normalized gene expression data of 17,419 genes (*Affymetrix Human Genome U219 Array*) was obtained from the GDSC database Release 8.3 (https://www.cancerrxgene.org/downloads/bulk_download).

#### Model inputs and outputs

We train multi-drug models that predict the relative inhibition for a given cell line being treated with given concentrations of one or more drug(s). The model inputs comprise cell line features based on gene expression, a representation of the applied drugs, and the corresponding drug concentrations. For the representation of drugs, we investigated four different settings, which are depicted in Figure 1 and will be described below.

To characterize cell lines in the model input, we performed a principal component analysis (PCA, R-package *stats* Version 3.6.3^57^) on the gene expression values and used the first 300 principal components (PCs) as cell line features. This dimension reduction method and feature number performed well in our recently published benchmarking of drug sensitivity prediction methods.^20^

In addition to the cell line features, we investigated four different settings for the encoding of drugs in the model input:

##### Setting 1 (OneHot)

In this setting, no drug properties are included. Instead, a 265-dimensional encoding of drugs is used. Each feature corresponds to one of the 265 drugs in our dataset. If a drug is part of the current entry, its feature is set to the corresponding log1p-normalized treatment concentration, otherwise it is set to 0.

##### Setting 2 (OneHotTar)

This setting uses the same concentration encoding as Setting 1 but additionally includes 290 drug target features. More precisely, we used the drug target annotations provided by DrugComb and included all molecules as targets that were targeted by at least five of the drugs in our dataset, resulting in a total of 290 target features. Each feature is then set to the number of drugs in the current entry that target the corresponding molecule (0, 1, or 2): Since DrugComb provides only data on monotherapies and two-drug combinations, the maximum value a target feature can have is 2, if it is targeted by both drugs in a two-drug combination entry. Note also that one drug can target more than one molecule.

##### Setting 3 (MACCS)

In this setting, each drug is represented by a 162-dimensional binary *molecular access system* (MACCS) fingerprint.^23^ Each position of the fingerprint corresponds to a molecular substructure, e.g., a functional group that may be present in a drug molecule. The respective bit is set to 1 if the corresponding substructure is present in the drug molecule at least once, and 0, otherwise. Additionally, one input feature for each drug is needed to denote its treatment concentration. Consequently, this setting uses a total of 2·162+2·1=326 drug features. To encode monotherapies, one of the fingerprints and the corresponding concentration are set to 0.

##### Setting 4 (PhysChem)

This setting is similar to Setting 3 but replaces each MACCS fingerprint with 182 numerical physico-chemical descriptors that denote different properties of the respective drugs, such as the molecular weight, number of valence electrons, or the logP value that measures lipophilicity. Consequently, this setting uses a total of 2·182+2·1=366 drug features. To denote monotherapies, one set of properties and the corresponding concentration are set to 0.

#### Machine learning algorithms

We investigate the predictive performance of three ML algorithms: neural networks random forests, and elastic net. We chose these models, since neural networks and tree-based methods are commonly used for synergy prediction.^7^ Furthermore, neural networks are also popular for drug sensitivity prediction,^42^^,^^58^^,^^59^ while random forest and elastic nets are used less frequently for this task.^29^^,^^32^^,^^60^^,^^61^^,^^62^^,^^63^ In our recently published benchmarking, we found, however, that tree-based methods and elastic nets frequently outperform neural networks in predicting drug responses.^20^ In line with our findings, several studies found that deep learning does not improve over conventional ML algorithms for making predictions on tabular data,^64^^,^^65^^,^^66^ or to generate feature representations for model inputs.^20^^,^^67^

All prediction models were implemented in Python 3.11. Random forests and elastic net models were implemented using *scikit-learn* Version 1.5.0,^53^ while neural networks were implemented using *tensorflow* Version 2.16.1^54^ with GPU support. The hyperparameters for each algorithm are provided in Tables S3.

#### Model training and testing

After filtering and processing the data as described above, we randomly divided the remaining cell lines into a training set (80% of cell lines) and a test set (20%). Since multiple data entries exist for each cell line (screening of different drugs/drug combinations at different concentrations), the final training data consists of all entries involving a cell line from the training set (3,741,209 entries). The final test data contains all remaining entries (1,550,215), i.e., all entries involving a cell line from the test set. This splitting ensures that the test performance is always evaluated on cell lines that were unseen during model training, thereby mimicking the scenario of making predictions for a previously unseen patient. In contrast, the same drugs and drug-combinations can occur in both the training and test data. To further ensure that the test cell lines do not affect the training process, the PCA-based input features of our models were computed using only cell lines from the training data.

On the training data, we performed a 5-fold cross validation (CV) to determine the best-performing hyperparameters of each ML model (see Table S4). The CV folds were generated by randomly dividing the training cell lines into five disjoint folds and assigning all entries involving a certain cell line to the corresponding fold. Since the number of available entries per cell line differs, the size of CV folds varies slightly between 644,308 and 857,361 entries. For the hyperparameter combination with smallest mean absolute error (MAE) averaged across all five folds, one final model is trained on the complete training data and its performance is evaluated on the test data.

During CV, we again recomputed the PCA-based cell line features using only the cell lines in the respective training folds.

For the models using one-hot encodings (Setting 1 and Setting 2), each drug has a designated input node. This is not the case for the models using drug features (Setting 3 and Setting 4), where swapping the features and concentration of the first drug with those of the second drug represents the same treatment but results in changes in the input representation (cf. input visualization in Figure 1). However, the model output should not depend on the order of the drugs in the input, i.e., it should not depend on whether drug features of a drug A in the input vector are located in front of or behind those of a drug B. Therefore, each original sample is included twice in the datasets for Settings 3 and 4. These duplicate samples differ only in the order of the drug features and concentrations: once in the order A-B, once in the order B-A. In the Results section, we investigate the impact on model performance when models are trained using the duplicated versus non-duplicated data. The test performance is always evaluated on the duplicated entries.

#### Dose-response curves and sensitivity measures

Using the relative inhibitions predicted by our models, it is possible to reconstruct dose-response curves for monotherapies and dose-response matrices for combination therapies (cf. Figure S25 for examples). Based on these curves/matrices, various measures of drug response can be derived. To this end, we first converted the (actual and predicted) relative inhibitions into relative viabilities by subtracting the relative inhibitions from 100 and dividing the result by 100. Additionally, we clamped viabilities to [0,1]. Note that we report relative viabilities in range [0,1] rather than range [0,100] to keep the results consistent and comparable to our previous study.^14^

To perform the curve-fitting for monotherapies, we employed a three-parametric logistic function from the *drc* R-package Version 3.0-1^55^^,^^68^:

$$f\left(x\right)=c+\frac{1-c}{1+\exp\left(b\cdot \left(\log\left(x\right)-\log\left(e\right)\right)\right)}$$

Here, f(x) denotes the estimated relative viability of the considered cell line at drug concentration *x*, *c* denotes the curve asymptote for increasing concentrations, *b* denotes the curve’s slope, and *e* denotes the concentration at the inflection point. We only fit curves when at least five dose-response points were available and we discarded all curves where the root mean squared error (RMSE) between the actual viabilities and those derived from the curve was greater than 0.3, a threshold that was previously employed for the data generation in the GDSC database.^33^^,^^34^

From the fitted curves, we derived two measures of monotherapy drug responses, namely IC50 values and CMax viabilities. The CMax viability is a novel drug sensitivity measure which we recently published.^14^ It is defined as the relative viability at the CMax concentration of the respective drug. The CMax concentration denotes the peak plasma concentration of a drug after administering the highest clinically recommended dose.^22^ Thus, the CMax viability is designed to estimate the maximal effect a treatment can realistically achieve. It ranges from 0 to 1, and smaller values denote a greater treatment effect. For the computation of CMax viabilities, we evaluated the function of the fitted curve at the drug’s CMax concentration. An exemplary visualization of the CMax viability can be found in Figure S25. For the computation of IC50 values, we intersected the dose-response curves with a horizontal line with y-intercept 0.5.

For combination therapies, we developed a variation of the CMax viability we call the *combination CMax viability* that can be derived from an actual/predicted dose-response matrix (cf. Figure S25). Our initial idea was to interpolate the values in the dose-response matrix to derive the relative viability when administering the CMax concentration of both combination drugs simultaneously. However, two synergistic drugs may have certain concentration windows with particularly high synergy/effectiveness.^69^ Thus, it is possible that the smallest viability is reached at a concentration combination smaller than the CMax concentrations. (Note that this should not happen for the dose-response curves we employed to compute the CMax viability for monotherapies since these curves are monotonically decreasing). Consequently, we considered the entire concentration range below the respective CMax values to compute our sensitivity measure. Conceptually, we want to derive the smallest viability within the area defined by the two concentration windows of the drugs limited at their respective CMax concentration. To compute the combination CMax viability, we linearly divided the concentration interval from 0 to the CMax for each drug into 100 equally spaced concentrations, each, resulting in 10,000 concentration combinations. For each combination, we estimated its relative viability through bilinear interpolation (R package *pracma*^70^ Version 2.4.2) from the full dose-response matrix. Finally, we define the minimum of all 10,000 values as the combination CMax viability. An exemplary visualization of this measure can be found in Figure S25.

As the CMax denotes the maximal feasible treatment concentration for a drug monotherapy, it may not be feasible to administer the CMax concentration of two drugs in combination. Yet, we believe that the respective CMax concentrations are a reasonable upper bound to consider for the computation of combination CMax viabilities. Note also that administering the CMax concentration for monotherapies might likewise not be feasible in all cases. Furthermore, the presented approach can theoretically be applied to any desired concentrations other than CMax.

### Quantification and statistical analysis

To assess the significance of performance differences between different prediction approaches (i.e., different ML algorithms and model inputs), we performed paired Wilcoxon signed rank tests^71^ using the R-package *stats* Version 3.6.3.^57^ To compare two approaches, we considered the absolute difference between the actual and predicted inhibition values for each test sample from both approaches. P-values were adjusted using the Bonferroni correction,^72^ where each p-value is multiplied by the number of tests. Here, we performed a pairwise comparison of n=12 approaches (3 ML algorithms, 4 input representations), resulting in (n·(n−1))/2=66 tests. All adjusted p-values <0.05 were considered significant.

Additionally, we computed effect sizes r∈[0,1] for each test using the R-package *rcompanion* Version 2.3.21.^73^ The absolute value of *r* indicates the strength of the effect, and the sign indicates the direction, i.e., which of the two compared methods resulted in lower absolute errors.
