# Supplementary material for: How to predict effective drug combinations – moving beyond synergy scores
Source: iScience. 2025 May 9;28(6):112622. doi: 10.1016/j.isci.2025.112622 (PMC12152377; doi:10.1016/j.isci.2025.112622)
Supplement: Document S1. Figures S1–S25, Tables S1–S4, Data S1, and Methods S1 [file mmc1.pdf]

iScience, Volume ■ ■

Supplemental information

How to predict effective drug combinations – moving beyond synergy scores

Lea Eckhart, Kerstin Lenhof, Lutz Herrmann, Lisa-Marie Rolli, and Hans-Peter Lenhof

Table S1: Drug sensitivity/synergy prediction literature, related to Introduction. This table lists 55 approaches for drug sensitivity or synergy prediction. For each approach, we denote (1) the predicted response (2) whether multi-drug models were trained (3) whether cell line-based omics features were employed (4) whether dose-specific predictions can be made (5) whether the approach explicitly employs dose-response curves/surfaces (6) whether a cell-blind evaluation was performed (7) whether drug- or combination-specific correlations are reported. Table continues on next pages.
[a] Post-treatment gene expression for dose-specific monotherapies is required such that doses are heavily constrained by data availability.

**Model (Year)**	**prediction**	**multi-drug**	**cell line features**	**dose-specific**	**curve- / surface-based**	**cell-blind**	**correl. per drug / combin.**
Menden et al. (2013) [1]	IC50	✓	✓	✗	✗	✗	✗
Pivetta et al. (2013) [2]	cytotoxicity, multi-drug effect index	(✓) two drugs	✗	✓	✓	✗	✗
Zhang et al. (2015) [3]	IC50, activity area	✓	✓	✗	✗	✗	✓
Gu et al. (2015) [4]	prostaglandin inhibition, combination index	✓	✗	✓	✗	(✗) one cell line	(✓) one cell line
DIGRE (2015) [5]	viability reduction	✓	✓	(✓)[a]	✓	(✗) two cell lines	✗
Rahman & Pal (2016) [6]	inhibition, AUC	✗	✓	✓	✓	✓	✓
Zimmer et al. (2016) [7]	viability	✓	✗	✓	✗	✗	✓
LOBICO (2016) [8]	binarized IC50	✗	✓	✗	✗	✓	✗
Hsu et al. (2016) [9]	own scores	✓	✓	✗	✗	(✓) no ML	✗
SRMF (2017) [10]	IC50, activity area	✓	✓	✗	✗	✗	✓
Zimmer et al. (2017) [11]	viability	✓	✗	✓	✗	✗	✗
SyDRa (2017) [12]	binarized synergy	✓	✓	(✓)[a]	✗	(✗) two cell lines	✗
Stanfield et al. (2017) [13]	binarized IC50	✓	✓	✗	✗	(✓) train on CCLE, test on GDSC	✗
HARF (2017) [14]	AUC	✗	✓	✗	✗	✓	✗
Jeon et al. (2017) [15]	(binarized) Loewe synergy score	✓	✓	✗	✗	✗	✗
TreeCombo (2018) [16]	Loewe synergy score	✗	✓	✗	✗	✗	✗
RWEN (2018) [17]	AUC	✗	✓	✗	✗	✓	✗
CDRscan (2018) [18]	IC50	✗	✓	✗	✗	✗	✓
QRF (2018) [19]	activity area	✗	✓	✗	✗	✓	✓
NCFGER (2018) [20]	IC50	✗	✓	✗	✗	✗	✓
TAIJI (2018) [21]	Loewe synergy score	✓	✓	✗	✓	? not stated	✗

Continuation of Table S1

**Model (Year)**	**prediction**	**multi-drug**	**cell line features**	**dose-specific**	**curve- / surface-based**	**cell-blind**	**Correl. per drug / combin.**
QPOP (2018) [22]	normalized viability	✓	✗	✓	✓	(✗) two cell lines	✗
Xia et al. (2018) [23]	growth percentage, modified ComboScore	✓	✓	✗	✗	✗	✗
HNMDRP (2018) [24]	IC50	✓	✓	✗	✗	✗	✗
Deep-Resp-Forest (2019) [25]	binarized IC50, binarized activity area	✗	✓	✗	✗	✓	✗
MOLI (2019) [26]	binarized IC50	(✓) drugs with same target pathway	✓	✗	✗	(✓) train on GDSC, test on TCGA + PDX	✗
DeepDR (2019) [27]	IC50	✓	✓	✗	✗	✓	✗
netBITE (2019) [28]	IC50	✗	✓	✗	✗	✓	✓
FRF (2019) [29]	dose-response distributions	✗	✓	✓	✓	✓	✗
Sidorov et al. (2019)[30]	ComboScore	(✓) cell line-specific models	✗	✗	✗	✗	✗
DECREASE (2019) [31]	relative inhibition, Bliss, Loewe, HSA, ZIP scores	✓	✗	(✓) only for inhibitions	✗	✗	✗
Deng et al. (2020) [32]	normalized AUC	✗	✓	✗	✗	(✓) LOOCV / train on CCLE, test on GDSC	✗
Ahmed et al. (2020) [33]	AUC, ED50	✗	✓	✗	✗	✓	✓
Ling & Huang (2020) [34]	averaged viability	✓	✗	✓	✗	✗	✓
ADRML (2020) [35]	IC50	✓	✓	✗	✗	✗	✓
Julkunen et al. (2020) [36]	cell growth, ComboScore	✓	✓	✓	✗	✗	✗
MinDrug (2021) [37]	IC50	(✓) subsets of similar drugs	✓	✗	✗	✓	✗
PathDSP (2021) [38]	IC50	✓	✓	✗	✗	✓	(✓) not cell-blind

Continuation of Table S1

**Model (Year)**	**prediction**	**multi-drug**	**cell line features**	**dose-specific**	**curve- / surface-based**	**cell-blind**	**Correl. per drug / combin.**
GraphDRP (2021) [39]	IC50	✓	✓	✗	✗	✓	✗
REFINED CNN (2021) [40]	normalized GI50, ComboScore	(✓) cell line-specific models	✓	✗	✗	✗	✗
Zheng et al. (2021) [41]	inhibition, Loewe, ZIP, HSA, Bliss synergy score	✓	✓	✓	✗	✗	✗
Correia et al. (2021) [42]	AUC for combinations	✓	✗	✓	✓	(✗) one cell line	✗
MERIDA (2021) [43]	binarized IC50	✗	✓	✗	✗	✓	✗
RAMP (2022) [44]	binarized IC50	✓	✓	✗	✗	(✓) patient data	✗
NeRD (2022) [45]	IC50	✓	✓	✗	✗	✓	✗
Precily (2022) [46]	IC50	✓	✓	✗	✗	✓	✓
Pinoli et al. (2022) [47]	ZIP synergy score	✓	✓	✗	✗	✓	✗
KBMTL (2014) [48]	AUC, discretized IC50	✓	✓	✗	✗	✓	✗
DeepSynergy (2018) [49]	Loewe synergy score	✓	✓	✗	✗	✗	✓
DeepCDR (2020) [50]	(binarized) IC50	✓	✓	✗	✗	✓	(✓) not cell-blind
Kim et al. (2021) [51]	(binarized) Loewe synergy score, (binarized) AUC	✓	✓	✗	✗	✗	✗
MatchMaker (2022) [52]	Loewe synergy score, ComboScore	✓	✓	✗	✗	✗	(✗) drug pairs
SAURON-RF (2022) [53]	(binarized) IC50	✗	✓	✗	✗	✓	(✓) only classification
reliable SAURON-RF (2023) [54]	(binarized) IC50, (binarized) CMax viability	✗	✓	✗	✗	✓	✓
GADRP (2023) [55]	normalized IC50	✓	✓	✗	✗	✗	✗
our approach	relative inhibition, IC50, (combination) CMax viability	✓	✓	✓	✗	✓	✓

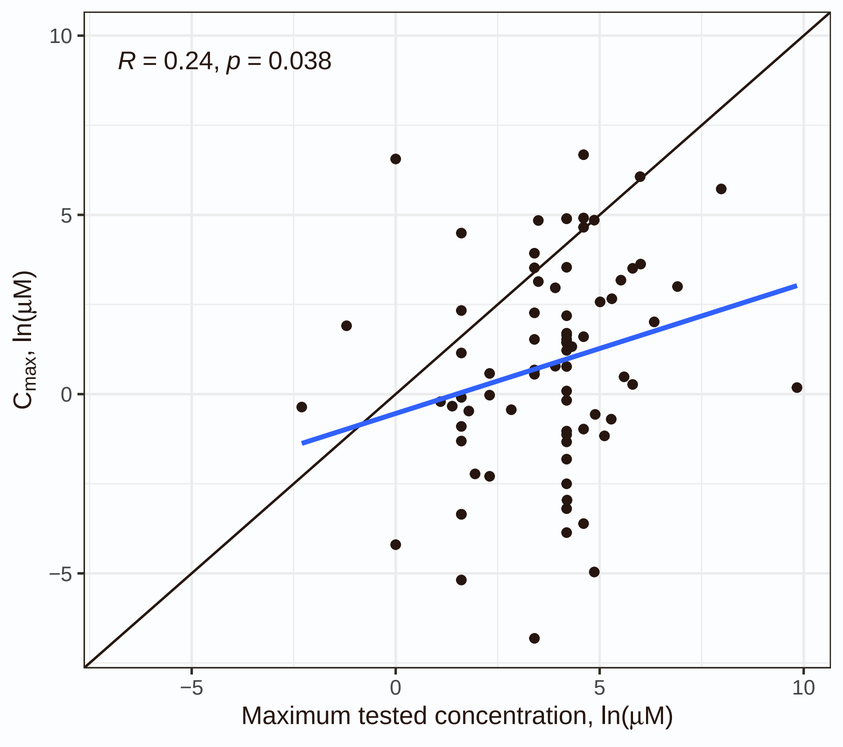

Figure S1: Comparison of CMax values and maximum tested drug concentrations, related to Section *Challenges of Synergy Scores for Recommending Personalized Treatments*. This figure depicts the CMax concentrations for 77 drugs from DrugComb in comparison to the maximum screened concentrations in our investigated dataset. The CMax concentrations were obtained from [56] and denote the peak plasma concentration of a drug after administering the highest clinically recommended dose. Additionally, the Pearson correlation coefficient (R) and a regression line (blue) are depicted.

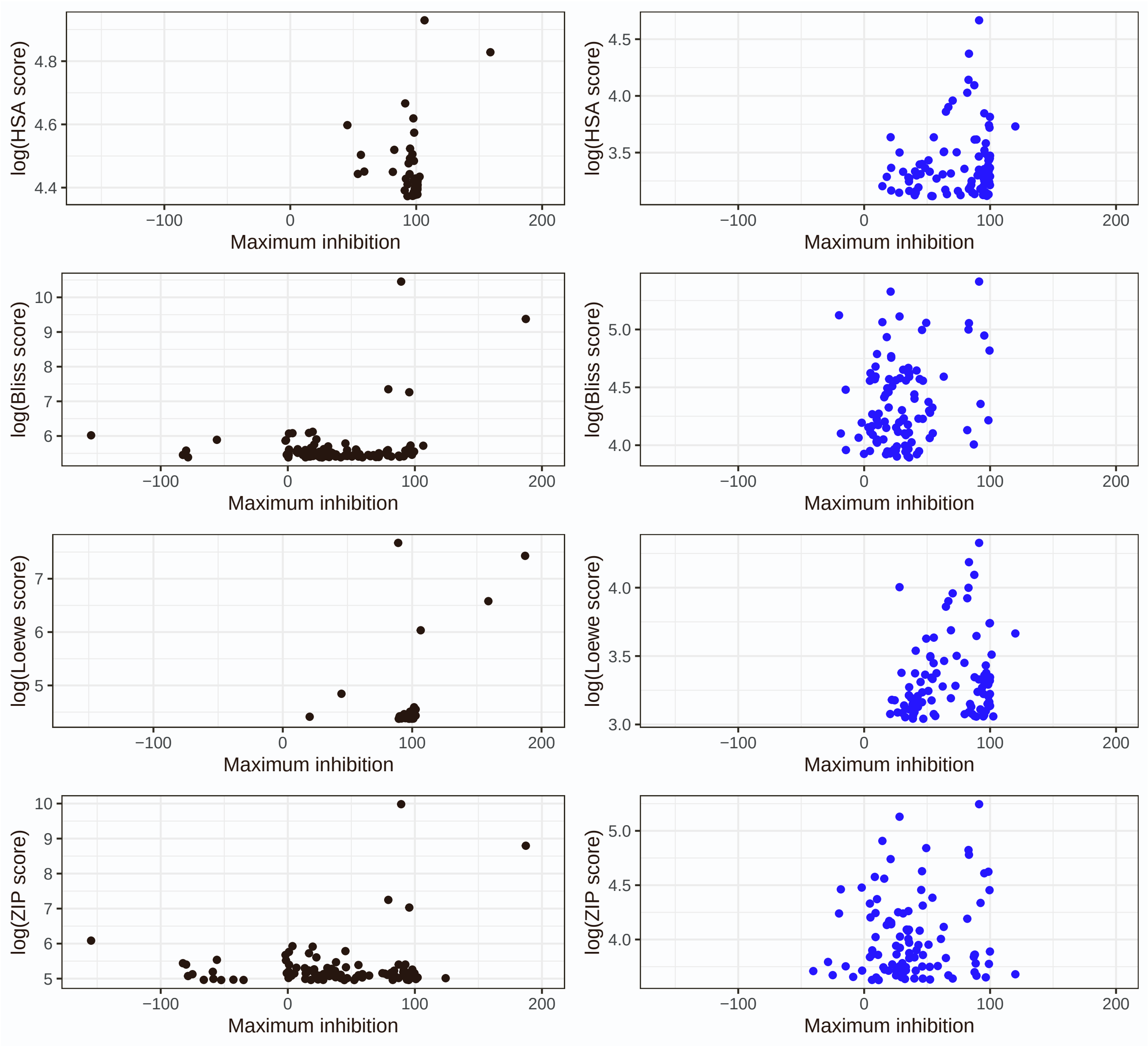

Figure S2: Comparison of synergy scores and maximum inhibition, related to section *Challenges of Synergy Scores for Recommending Personalized Treatments*. For each synergy score (HSA, Bliss, Loewe, ZIP), the 100 experiments with the highest score are depicted. The y-axis shows the respective score, and the x-axis shows the maximum relative inhibition reached in the respective experiment. The plots on the left (black dots) show the results for all experiments in DrugComb. The plots on the right (blue dots) show the results for those 77 drugs in our analysis, for which we could obtain the CMax concentration from Liston and Davis [56]. Additionally, for the plots on the right, we considered only those experiments where concentrations exceeding CMax were screened for both drugs to ensure that sufficiently large concentrations were tested. Note that these plots were generated without the data processing discussed in the STAR Methods (e.g., aggregation of replicates). We solely discarded entries with relative inhibition > 200 or < -200.

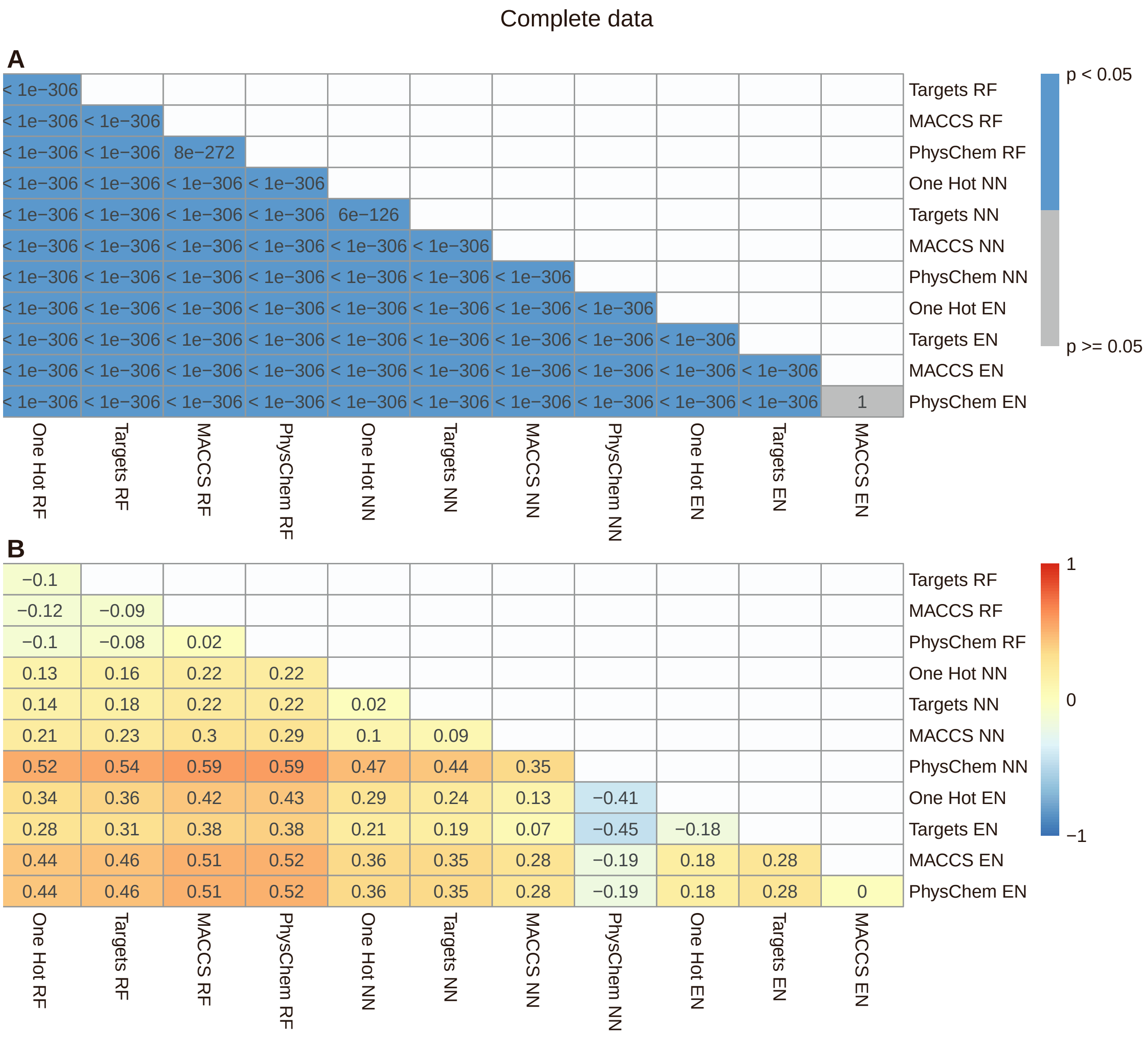

Figure S3: Statistical comparison of models using the complete test data, related to Figure 2. Sub-figure A shows the Bonferroni-adjusted p-values when comparing the test set predictions of two models using paired Wilcoxon signed rank tests. Sub-figure B shows the corresponding effect sizes $r \in [-1, 1]$. The absolute value of r indicates the strength of the effect, and the sign indicates the direction. For $r > 0$, the method specified in the respective column outperformed the method specified in the respective row.

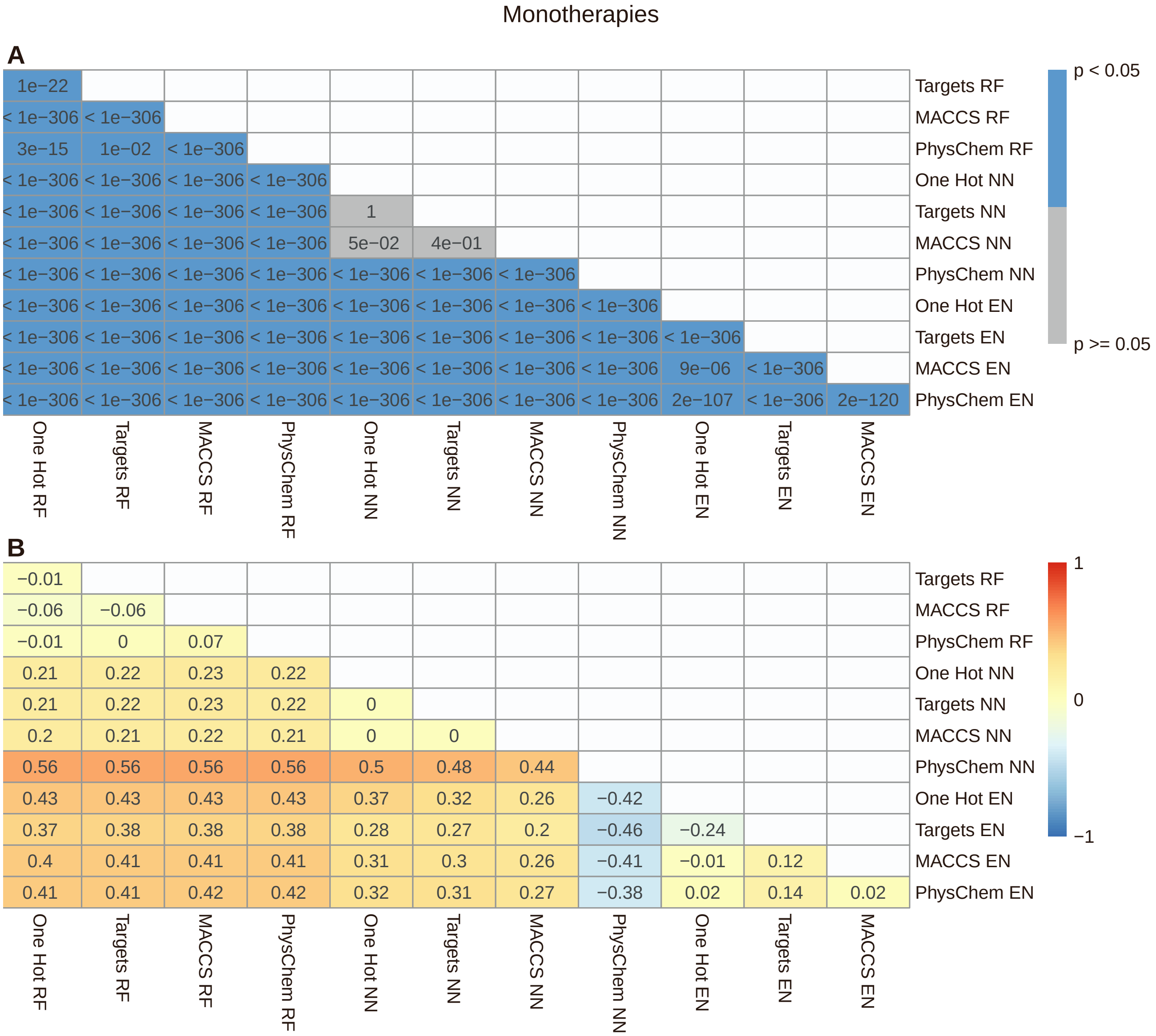

Figure S4: Statistical comparison of models using the monotherapy test data, related to Figure 2. Sub-figure A shows the Bonferroni-adjusted p-values when comparing the test set predictions of two models using paired Wilcoxon signed rank tests. Sub-figure B shows the corresponding effect sizes $r \in [-1, 1]$. The absolute value of r indicates the strength of the effect, and the sign indicates the direction. For $r > 0$, the method specified in the respective column outperformed the method specified in the respective row.

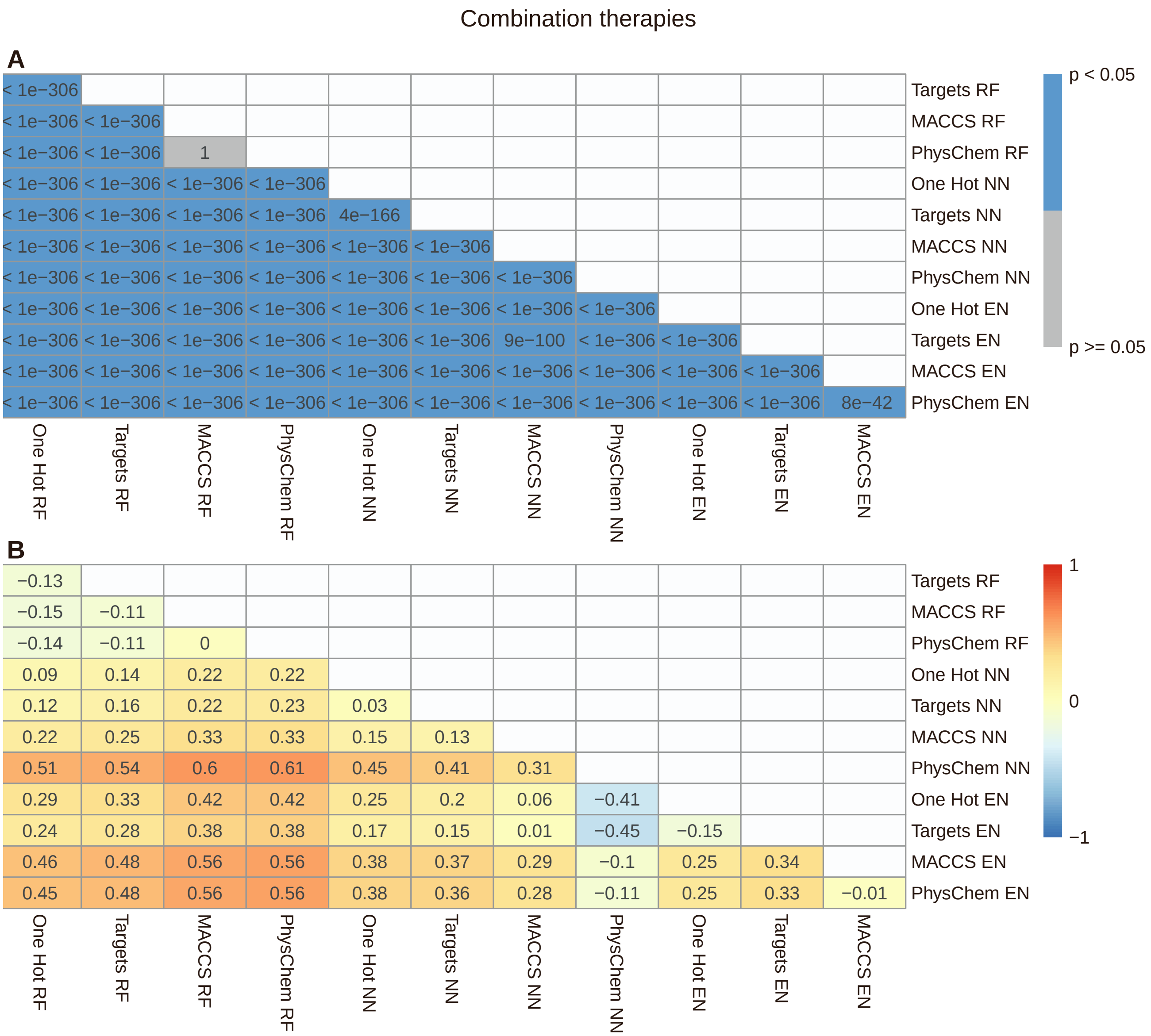

Figure S5: Statistical comparison of models using the combination therapy test data, related to Figure 2. Sub-figure A shows the Bonferroni-adjusted p-values when comparing the test set predictions of two models using paired Wilcoxon signed rank tests. Sub-figure B shows the corresponding effect sizes $r \in [-1, 1]$. The absolute value of r indicates the strength of the effect, and the sign indicates the direction. For $r > 0$, the method specified in the respective column outperformed the method specified in the respective row.

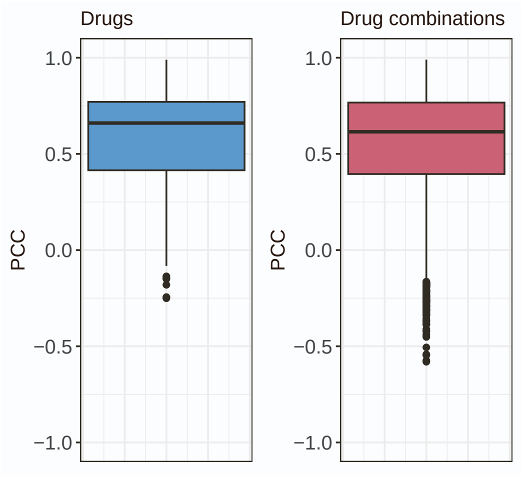

Figure S6: Average Pearson correlations between actual and predicted relative inhibitions per drug (blue) and per drug combination (red) using the MACCS random forest, related to Figure 2. Data are represented as boxplots where the box denotes the interquartile range between the first quartile (25th percentile) and third quartile (75th percentile) of the data. The black horizontal line inside each box denotes the median, and the whiskers extend to the largest/smallest values within 1.5 times the interquartile range.

Complete data

	One Hot RF	Targets RF	MACCS dup. RF	PhysChem dup. RF	One Hot NN	Targets NN	MACCS dup. NN	PhysChem dup. NN	One Hot EN	Targets EN	MACCS dup. EN	PhysChem dup. EN
PCC total	0.76	0.78	0.8	0.8	0.69	0.7	0.72	0.18	0.57	0.63	0.48	0.49
PCC Monotherapies	0.77	0.77	0.77	0.77	0.71	0.71	0.72	0.17	0.58	0.63	0.55	0.54
PCC Combination Therapies	0.76	0.78	0.82	0.82	0.68	0.69	0.72	0.17	0.57	0.63	0.46	0.46
Avg. PCC per Drug in Monotherapies	0.55	0.56	0.58	0.57	0.47	0.47	0.49	0.07	0.37	0.39	0.42	0.42
Avg. PCC per Combination	0.48	0.51	0.56	0.56	0.44	0.39	0.45	0.04	0.34	0.37	0.36	0.36
R2 total	0.58	0.6	0.65	0.65	0.48	0.48	0.52	0.03	0.33	0.4	0.23	0.24
R2 Monotherapies	0.59	0.59	0.6	0.59	0.5	0.5	0.52	0.03	0.34	0.39	0.31	0.29
R2 Combination Therapies	0.58	0.61	0.67	0.67	0.47	0.48	0.52	0.03	0.33	0.4	0.22	0.21
Avg. R2 per Drug in Monotherapies	0.38	0.39	0.41	0.4	0.31	0.31	0.33	0.03	0.24	0.25	0.25	0.25
Avg. R2 per Combination	0.32	0.34	0.39	0.39	0.29	0.27	0.29	0.02	0.23	0.24	0.19	0.18

Color scale: 1, 0.5, 0

Figure S7: Pearson correlation coefficients (PCC) and coefficient of determination (R2) values for the test set, related to Figure 2. The PCC/R2 values are shown for the total test dataset, as well as for the subsets of mono- and combination therapies. Additionally, the average PCC/R2 per drug for monotherapies and the average PCC/R2 per drug combination for combination therapies are provided.

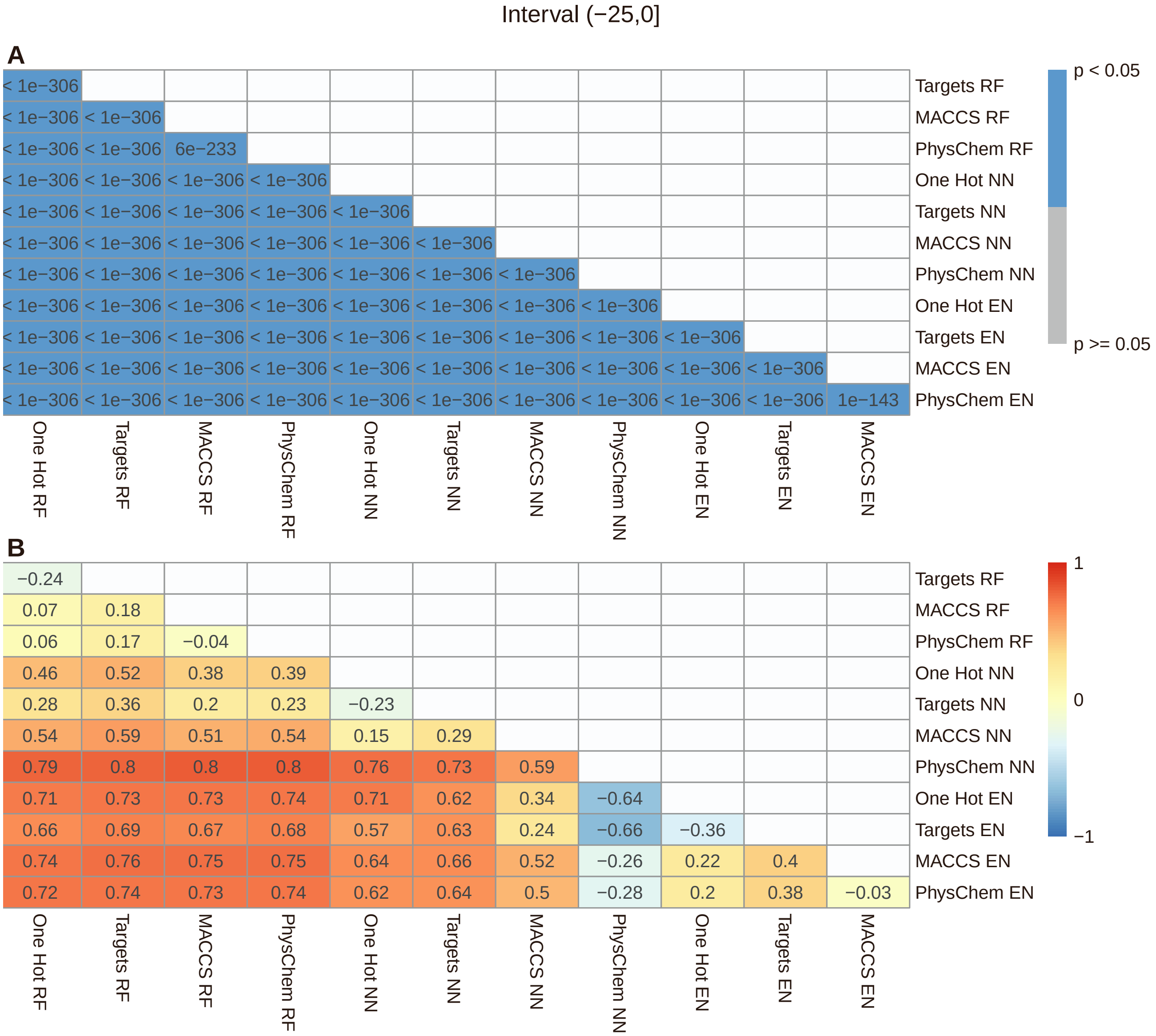

Figure S8: Statistical comparison of models using the test data with relative inhibition in $(-25, 0]$, related to Figure 3. Sub-figure A shows the Bonferroni-adjusted p-values when comparing the test set predictions of two models using paired Wilcoxon signed rank tests. Sub-figure B shows the corresponding effect sizes $r \in [-1, 1]$. The absolute value of r indicates the strength of the effect, and the sign indicates the direction. For $r > 0$, the method specified in the respective column outperformed the method specified in the respective row.

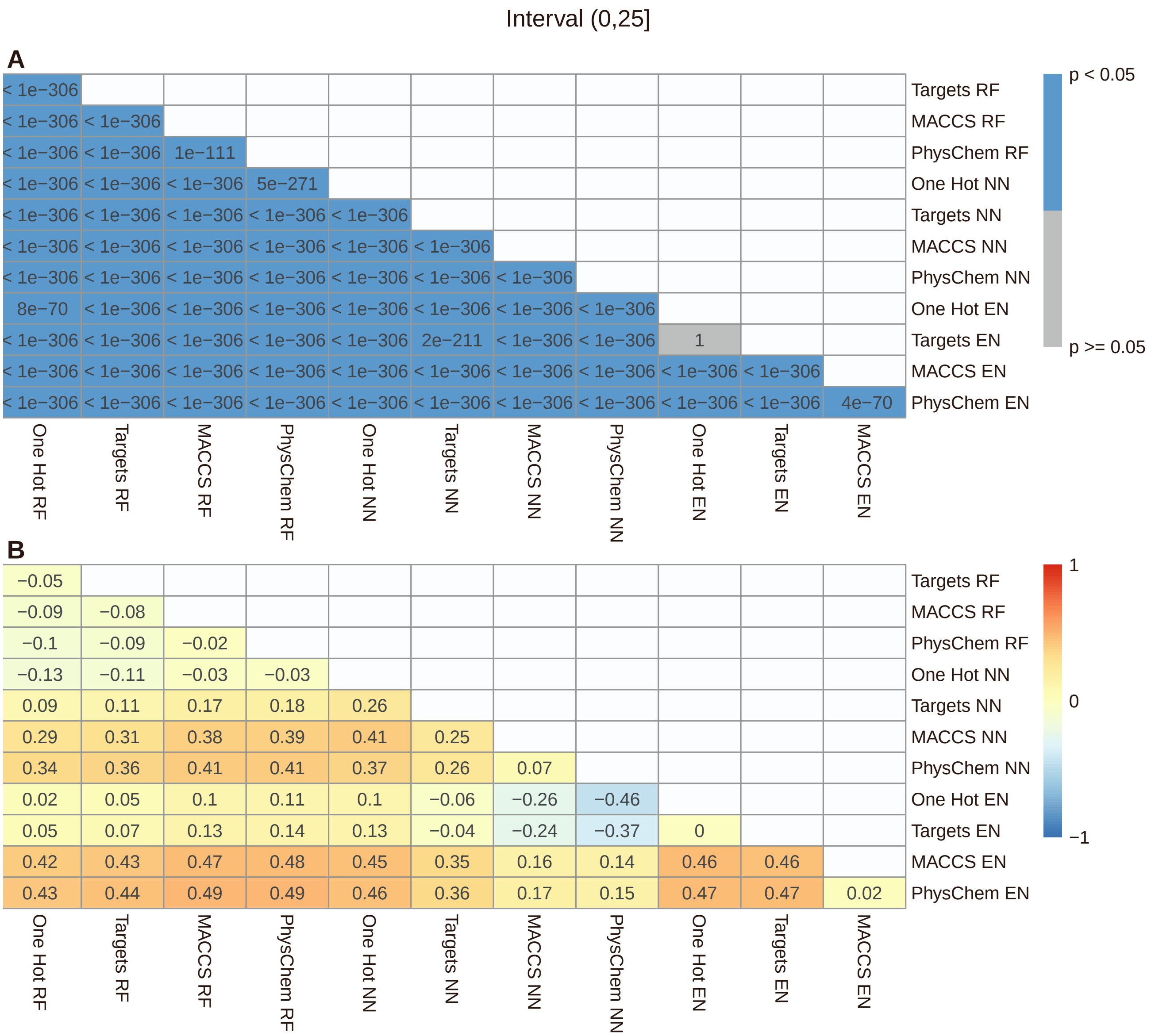

Figure S9: Statistical comparison of models using the test data with relative inhibition in $(0, 25]$, related to Figure 3. Sub-figure A shows the Bonferroni-adjusted p-values when comparing the test set predictions of two models using paired Wilcoxon signed rank tests. Sub-figure B shows the corresponding effect sizes $r \in [-1, 1]$. The absolute value of r indicates the strength of the effect, and the sign indicates the direction. For $r > 0$, the method specified in the respective column outperformed the method specified in the respective row.

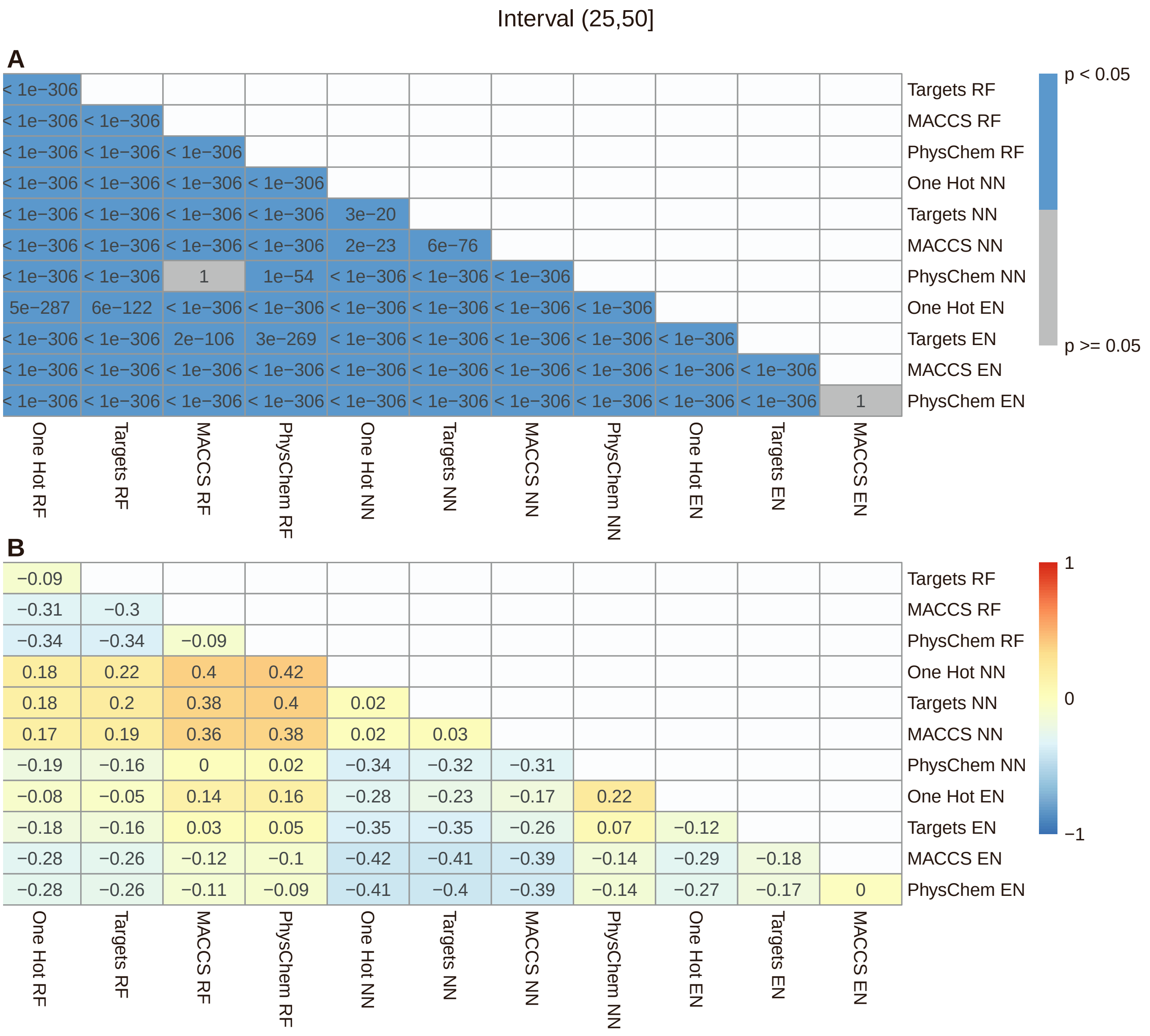

Figure S10: Statistical comparison of models using the test data with relative inhibition in $(25, 50]$, related to Figure 3. Sub-figure A shows the Bonferroni-adjusted p-values when comparing the test set predictions of two models using paired Wilcoxon signed rank tests. Sub-figure B shows the corresponding effect sizes $r \in [-1, 1]$. The absolute value of r indicates the strength of the effect, and the sign indicates the direction. For $r > 0$, the method specified in the respective column outperformed the method specified in the respective row.

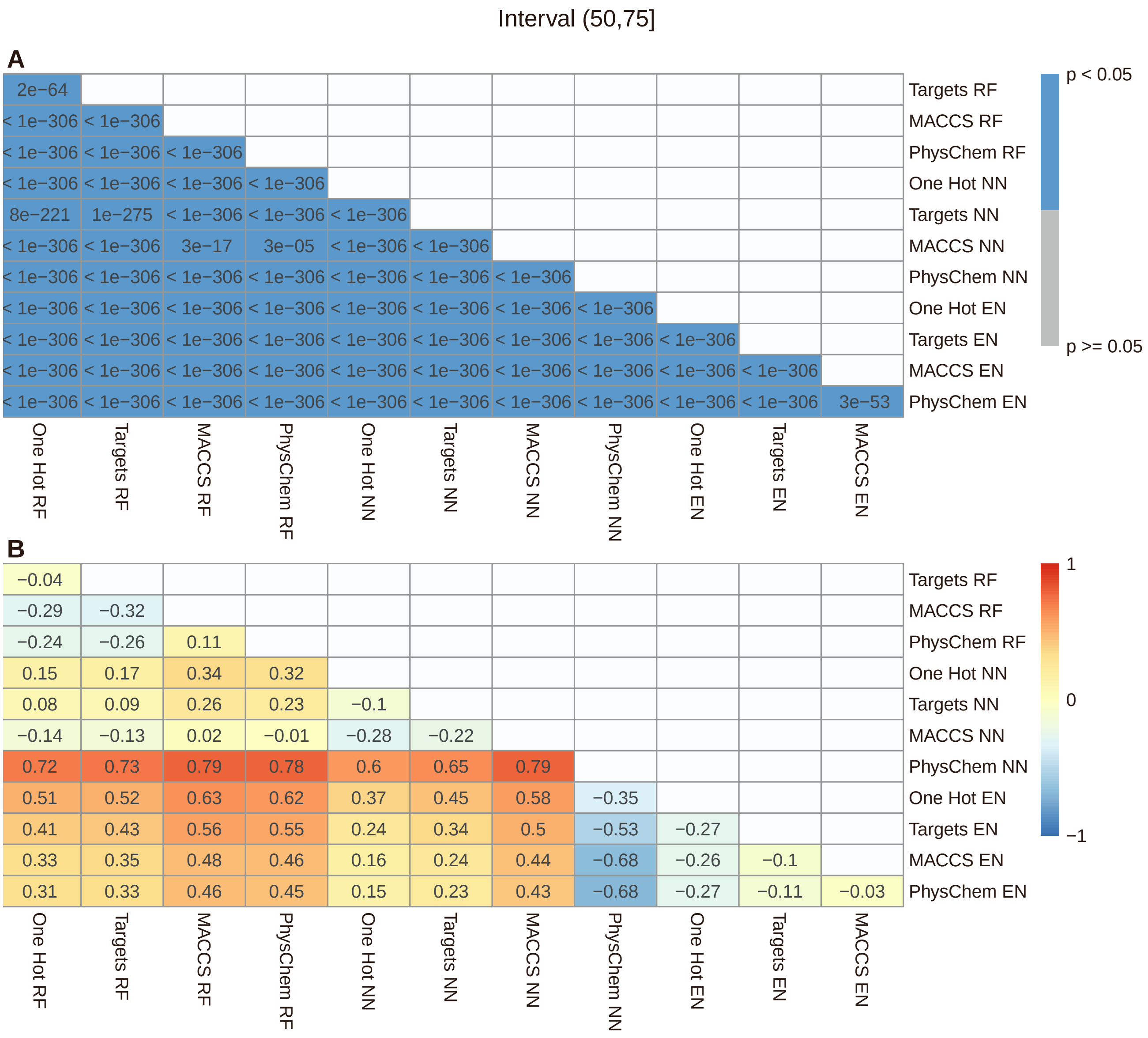

Figure S11: Statistical comparison of models using the test data with relative inhibition in $(50, 75]$, related to Figure 3. Sub-figure A shows the Bonferroni-adjusted p-values when comparing the test set predictions of two models using paired Wilcoxon signed rank tests. Sub-figure B shows the corresponding effect sizes $r \in [-1, 1]$. The absolute value of r indicates the strength of the effect, and the sign indicates the direction. For $r > 0$, the method specified in the respective column outperformed the method specified in the respective row.

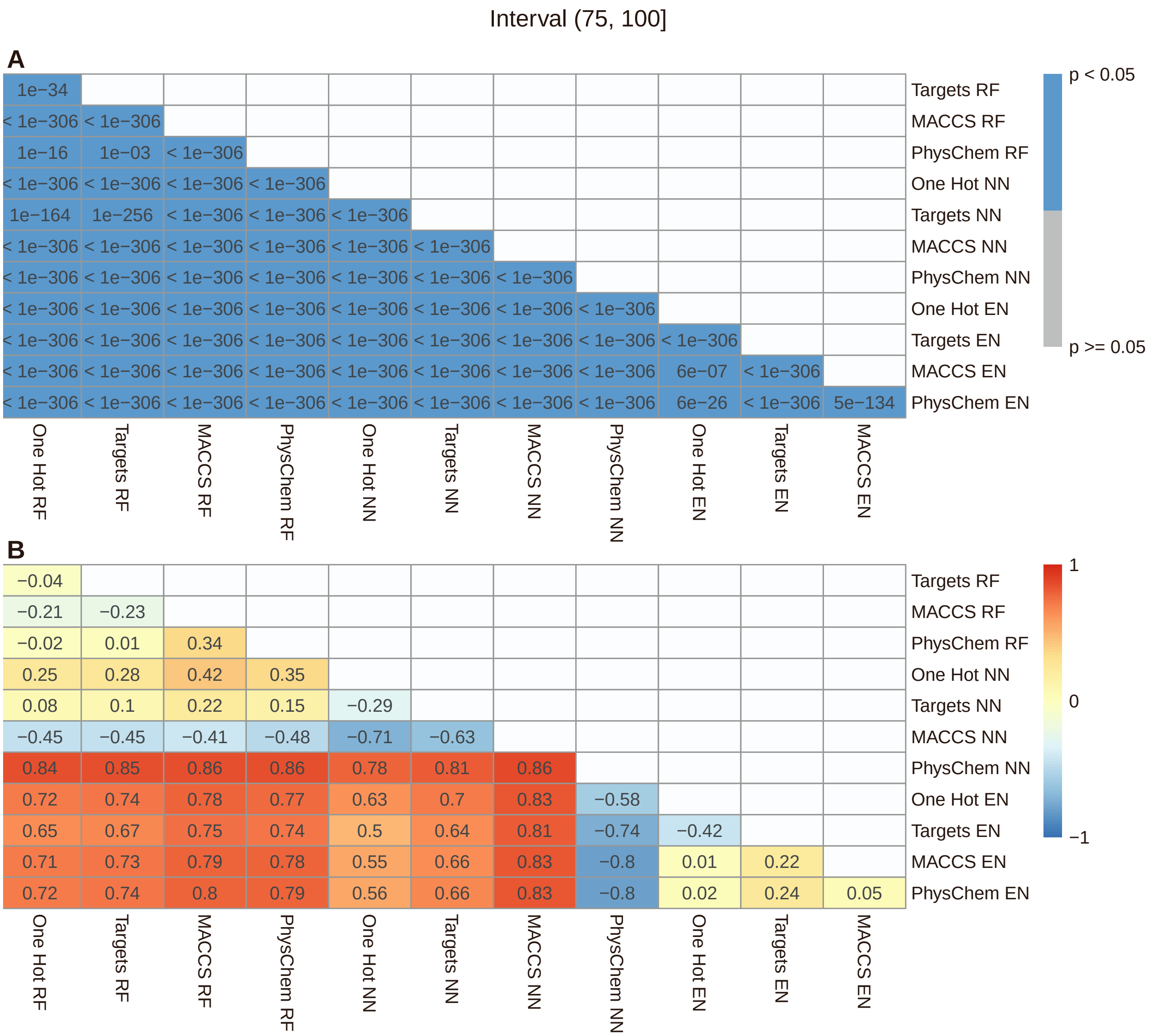

Figure S12: Statistical comparison of models using the test data with relative inhibition in $(75, 100]$, related to Figure 3. Sub-figure A shows the Bonferroni-adjusted p-values when comparing the test set predictions of two models using paired Wilcoxon signed rank tests. Sub-figure B shows the corresponding effect sizes $r \in [-1, 1]$. The absolute value of r indicates the strength of the effect, and the sign indicates the direction. For $r > 0$, the method specified in the respective column outperformed the method specified in the respective row.

Interval (−25,0]

	One Hot RF	Targets RF	MACCS dup. RF	PhysChem dup. RF	One Hot NN	Targets NN	MACCS dup. NN	PhysChem dup. NN	One Hot EN	Targets EN	MACCS dup. EN	PhysChem dup. EN
PCC total	0.02	0.02	0.04	0.04	−0.01	0.01	0.02	−0.01	−0.03	−0.02	0.03	0.03
PCC Monotherapies	0.01	0.01	0.02	0.02	−0.01	0	0	−0.01	0.01	0.01	0.01	0
PCC Combination Therapies	0.01	0.01	0.03	0.04	0	0.01	0	−0.04	−0.04	−0.02	−0.02	−0.01
Avg. PCC per Drug in Monotherapies	−0.01	−0.01	−0.01	−0.01	−0.03	−0.02	−0.02	−0.01	−0.02	0	−0.01	−0.01
Avg. PCC per Combination	0.02	0.01	0.01	0.02	−0.02	0	−0.01	−0.01	−0.03	−0.05	−0.03	−0.03
R2 total	0	0	0	0	0	0	0	0	0	0	0	0
R2 Monotherapies	0	0	0	0	0	0	0	0	0	0	0	0
R2 Combination Therapies	0	0	0	0	0	0	0	0	0	0	0	0
Avg. R2 per Drug in Monotherapies	0.06	0.06	0.05	0.06	0.06	0.05	0.05	0.01	0.06	0.06	0.07	0.06
Avg. R2 per Combination	0.16	0.16	0.14	0.13	0.16	0.16	0.13	0.01	0.16	0.16	0.17	0.17

Figure S13: Pearson correlation coefficients (PCC) and coefficient of determination (R2) values for the test samples in the interval $(-25, 0]$, related to Figure 3. The PCC/R2 values are shown for the total test dataset within the range $(-25, 0]$, as well as for the subsets of mono- and combination therapies. Additionally, the average PCC/R2 per drug for monotherapies and the average PCC/R2 per drug combination for combination therapies are provided.

Interval (0,25]

	One Hot RF	Targets RF	MACCS dup. RF	PhysChem dup. RF	One Hot NN	Targets NN	MACCS dup. NN	PhysChem dup. NN	One Hot EN	Targets EN	MACCS dup. EN	PhysChem dup. EN
PCC total	0.32	0.34	0.4	0.4	0.28	0.27	0.31	0.1	0.25	0.27	0.2	0.2
PCC Monotherapies	0.33	0.33	0.33	0.33	0.28	0.28	0.3	0.1	0.21	0.25	0.22	0.21
PCC Combination Therapies	0.32	0.34	0.43	0.43	0.28	0.26	0.32	0.1	0.26	0.28	0.19	0.2
Avg. PCC per Drug in Monotherapies	0.31	0.32	0.34	0.34	0.24	0.25	0.28	0.05	0.17	0.19	0.23	0.23
Avg. PCC per Combination	0.26	0.28	0.32	0.33	0.25	0.21	0.26	0.02	0.2	0.23	0.23	0.23
R2 total	0.11	0.12	0.16	0.16	0.08	0.07	0.1	0.01	0.06	0.07	0.04	0.04
R2 Monotherapies	0.11	0.11	0.11	0.11	0.08	0.08	0.09	0.01	0.04	0.06	0.05	0.05
R2 Combination Therapies	0.1	0.12	0.18	0.18	0.08	0.07	0.1	0.01	0.07	0.08	0.04	0.04
Avg. R2 per Drug in Monotherapies	0.14	0.15	0.16	0.15	0.1	0.1	0.12	0.02	0.08	0.09	0.1	0.1
Avg. R2 per Combination	0.14	0.15	0.17	0.17	0.13	0.12	0.13	0.01	0.11	0.12	0.11	0.11

Figure S14: Pearson correlation coefficients (PCC) and coefficient of determination (R2) values for the test samples in the interval $(0, 25]$, related to Figure 3. The PCC/R2 values are shown for the total test dataset within the range $(0, 25]$, as well as for the subsets of mono- and combination therapies. Additionally, the average PCC/R2 per drug for monotherapies and the average PCC/R2 per drug combination for combination therapies are provided.

Interval (25,50]

	One Hot RF	Targets RF	MACCS dup. RF	PhysChem dup. RF	One Hot NN	Targets NN	MACCS dup. NN	PhysChem dup. NN	One Hot EN	Targets EN	MACCS dup. EN	PhysChem dup. EN
PCC total	0.25	0.26	0.28	0.27	0.2	0.21	0.22	0.05	0.17	0.19	0.12	0.12
PCC Monotherapies	0.24	0.24	0.25	0.24	0.21	0.21	0.21	0.04	0.16	0.18	0.16	0.15
PCC Combination Therapies	0.26	0.26	0.29	0.29	0.2	0.21	0.23	0.05	0.17	0.19	0.11	0.11
Avg. PCC per Drug in Monotherapies	0.17	0.17	0.14	0.15	0.14	0.11	0.14	0.02	0.14	0.14	0.13	0.13
Avg. PCC per Combination	0.17	0.19	0.22	0.22	0.15	0.13	0.16	0.01	0.12	0.13	0.12	0.12
R2 total	0.06	0.07	0.08	0.08	0.04	0.04	0.05	0	0.03	0.03	0.01	0.01
R2 Monotherapies	0.06	0.06	0.06	0.06	0.04	0.04	0.05	0	0.03	0.03	0.02	0.02
R2 Combination Therapies	0.07	0.07	0.09	0.09	0.04	0.04	0.05	0	0.03	0.04	0.01	0.01
Avg. R2 per Drug in Monotherapies	0.13	0.12	0.1	0.09	0.11	0.11	0.1	0.01	0.12	0.11	0.1	0.1
Avg. R2 per Combination	0.2	0.2	0.19	0.19	0.19	0.18	0.16	0.01	0.18	0.18	0.16	0.17

Figure S15: Pearson correlation coefficients (PCC) and coefficient of determination (R2) values for the test samples in the interval $(25, 50]$, related to Figure 3. The PCC/R2 values are shown for the total test dataset within the range $(25, 50]$, as well as for the subsets of mono- and combination therapies. Additionally, the average PCC/R2 per drug for monotherapies and the average PCC/R2 per drug combination for combination therapies are provided.

Interval (50,75]

	One Hot RF	Targets RF	MACCS dup. RF	PhysChem dup. RF	One Hot NN	Targets NN	MACCS dup. NN	PhysChem dup. NN	One Hot EN	Targets EN	MACCS dup. EN	PhysChem dup. EN
PCC total	0.21	0.21	0.21	0.22	0.17	0.18	0.17	0	0.14	0.16	0.09	0.09
PCC Monotherapies	0.18	0.18	0.18	0.18	0.15	0.15	0.16	0	0.1	0.12	0.09	0.09
PCC Combination Therapies	0.21	0.22	0.23	0.23	0.18	0.18	0.19	0	0.15	0.17	0.1	0.1
Avg. PCC per Drug in Monotherapies	0.15	0.11	0.15	0.13	0.1	0.1	0.11	0	0.12	0.11	0.12	0.12
Avg. PCC per Combination	0.13	0.13	0.16	0.16	0.13	0.11	0.13	0.01	0.08	0.09	0.1	0.1
R2 total	0.04	0.04	0.05	0.05	0.03	0.03	0.03	0	0.02	0.02	0.01	0.01
R2 Monotherapies	0.03	0.03	0.03	0.03	0.02	0.02	0.02	0	0.01	0.01	0.01	0.01
R2 Combination Therapies	0.05	0.05	0.05	0.05	0.03	0.03	0.03	0	0.02	0.03	0.01	0.01
Avg. R2 per Drug in Monotherapies	0.15	0.15	0.12	0.12	0.14	0.13	0.13	0.02	0.15	0.15	0.15	0.15
Avg. R2 per Combination	0.26	0.26	0.22	0.22	0.26	0.26	0.19	0.02	0.25	0.24	0.23	0.23

Figure S16: Pearson correlation coefficients (PCC) and coefficient of determination (R2) values for the test samples in the interval $(50, 75]$, related to Figure 3. The PCC/R2 values are shown for the total test dataset within the range $(50, 75]$, as well as for the subsets of mono- and combination therapies. Additionally, the average PCC/R2 per drug for monotherapies and the average PCC/R2 per drug combination for combination therapies are provided.

Interval (75, 100]

	One Hot RF	Targets RF	MACCS dup. RF	PhysChem dup. RF	One Hot NN	Targets NN	MACCS dup. NN	PhysChem dup. NN	One Hot EN	Targets EN	MACCS dup. EN	PhysChem dup. EN
PCC total	0.24	0.26	0.28	0.29	0.15	0.17	0.19	−0.01	0.14	0.16	0.14	0.13
PCC Monotherapies	0.34	0.34	0.35	0.34	0.28	0.29	0.28	0	0.23	0.24	0.21	0.21
PCC Combination Therapies	0.17	0.2	0.23	0.25	0.07	0.11	0.15	0	0.06	0.09	0.18	0.13
Avg. PCC per Drug in Monotherapies	0.16	0.14	0.14	0.15	0.12	0.15	0.15	0.01	0.16	0.16	0.16	0.16
Avg. PCC per Combination	0.12	0.13	0.13	0.14	0.09	0.06	0.08	0.01	0.07	0.08	0.13	0.13
R2 total	0.06	0.07	0.08	0.08	0.02	0.03	0.04	0	0.02	0.03	0.02	0.02
R2 Monotherapies	0.12	0.12	0.12	0.12	0.08	0.08	0.08	0	0.05	0.06	0.04	0.04
R2 Combination Therapies	0.03	0.04	0.05	0.06	0	0.01	0.02	0	0	0.01	0.03	0.02
Avg. R2 per Drug in Monotherapies	0.16	0.16	0.14	0.15	0.15	0.14	0.15	0.02	0.16	0.15	0.15	0.15
Avg. R2 per Combination	0.32	0.32	0.23	0.23	0.3	0.31	0.21	0.02	0.29	0.29	0.28	0.28

Colour scale: 1, 0.5, 0

Figure S17: Pearson correlation coefficients (PCC) and coefficient of determination (R2) values for the test samples in the interval $(75, 100]$, related to Figure 3. The PCC/R2 values are shown for the total test dataset within the range $(75, 100]$, as well as for the subsets of mono- and combination therapies. Additionally, the average PCC/R2 per drug for monotherapies and the average PCC/R2 per drug combination for combination therapies are provided.

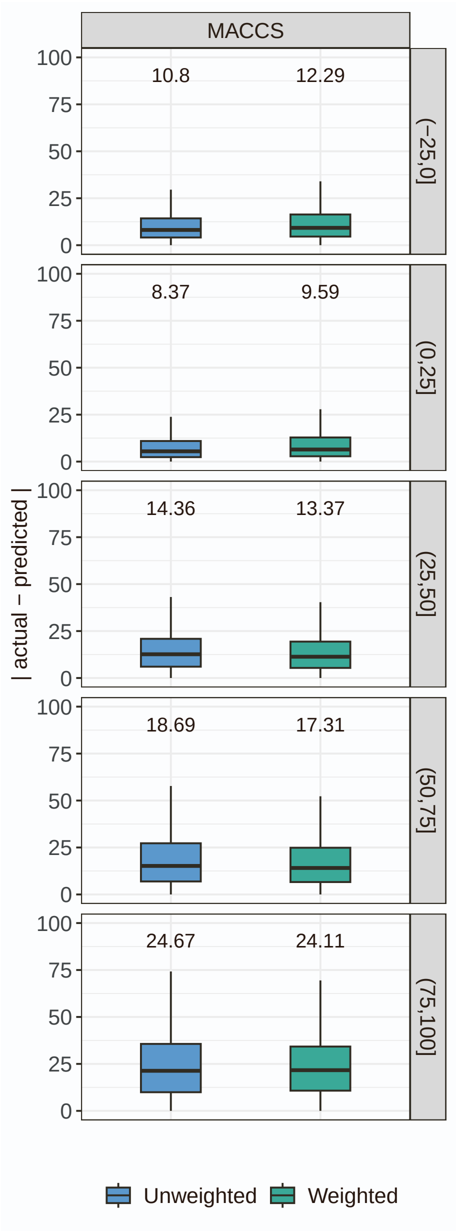

Figure S18: Impact of sample weights on model predictions, related to Figure 3. This figure compares the prediction performance (in terms of absolute difference between actual and predicted values) for the random forest MACCS model with (green) and without (blue) sample weights. Each row shows the performance for a different interval of actual relative inhibitions. Data are represented as boxplots where the box denotes the interquartile range between the first quartile (25th percentile) and third quartile (75th percentile) of the data. The black horizontal line inside each box denotes the median, and the whiskers extend to the largest/smallest values within 1.5 times the interquartile range. Outliers are not shown. On top of each boxplot, the mean absolute error (MAE) is shown. The weight of each sample was determined based on its interval $i \in I = \{(-\infty, 0], (0, 25], (25, 50], (50, 75], (75, \infty)\}$ as $(\frac{max_{j \in I}|j|}{|i|})^2$, where $|i|$ denotes the number of training samples in interval i.

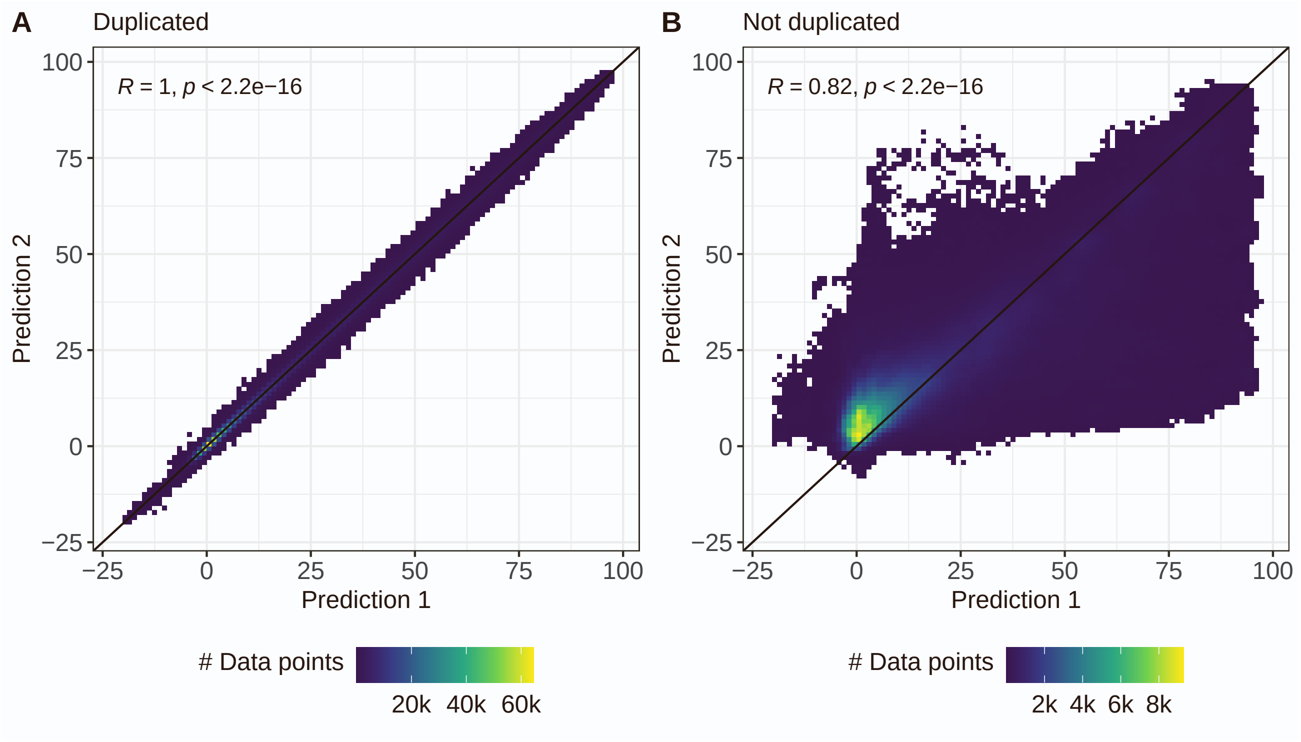

Figure S19: Correlation of duplicated entries from the test data, related to Figure 4. This figure shows the correlation between the predictions for duplicated entries obtained from the random forest PhysChem model. Duplicated entries refer to the same drug-drug-cell combination and the same treatment concentrations but can be represented by two different model inputs through swapping the features of the respective drugs (cf. Methods and Figure 1 in the main manuscript). Sub-figure A shows the test predictions when including duplicated entries into the training data, while Sub-figure B shows the predictions when training only on non-duplicated entries. In both figures, the black diagonal line represents the identity and R denotes the Pearson correlation between the predictions.

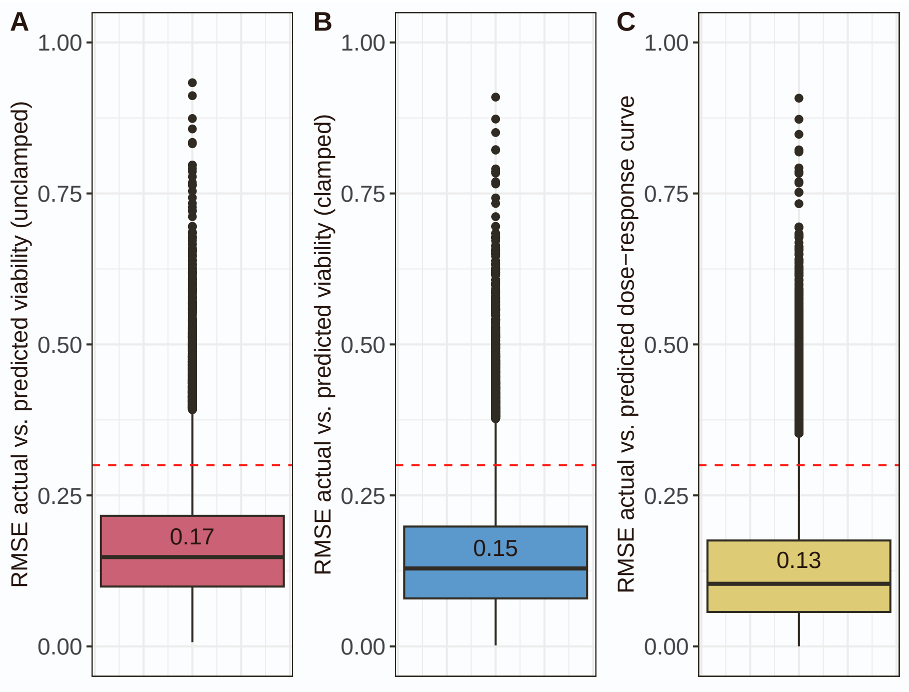

Figure S20: Reconstruction of dose-response curves, related to Figure 5. This figure shows the RMSE of reconstructing monotherapy dose-response curves from the test set using the MACCS random forest model. Sub-figure A shows the RMSE between the actual and predicted viabilities for each cell line-drug combination (i.e., each dose-response curve) in the test data. Sub-figure B shows the same data but viabilities are clamped to range $[0, 1]$. Sub-figure C shows the RMSE between the corresponding points on the actual and predicted dose-response curve. Data are represented as boxplots where the box denotes the interquartile range between the first quartile (25th percentile) and third quartile (75th percentile) of the data. The black horizontal line inside each box denotes the median, and the whiskers extend to the largest/smallest values within 1.5 times the interquartile range. Inside each boxplot, the mean absolute error (MAE) is shown. The red dashed line in each plot corresponds to an RMSE of 0.3, a threshold used in the GDSC database to identify poor-quality curves [57, 58].

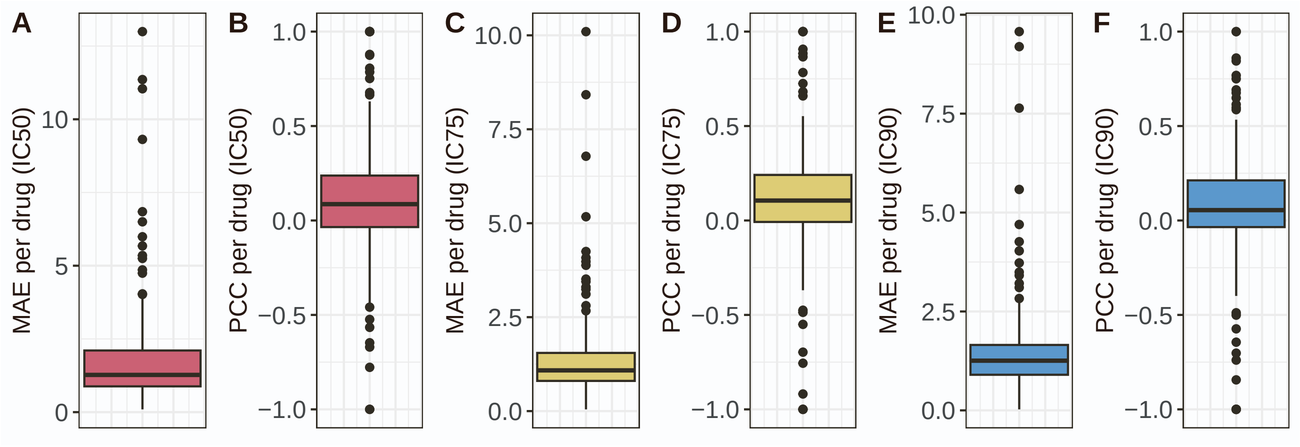

Figure S21: Reconstruction of IC50, IC75, and IC90 values from model predictions, related to Figure 5. Sub-figures A and B (red) show the distribution of MAE and PCC per drug for the reconstruction of IC50 values using the test set monotherapy data. Sub-figures C and D (yellow) show the analogous results for IC75 values. Sub-figures E and F (blue) show the analogous results for IC90 values. Data are represented as boxplots where the box denotes the interquartile range between the first quartile (25th percentile) and third quartile (75th percentile) of the data. The black horizontal line inside each box denotes the median, and the whiskers extend to the largest/smallest values within 1.5 times the interquartile range.

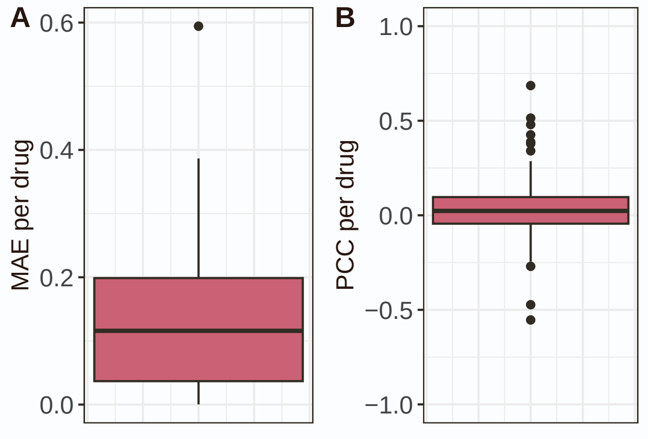

Figure S22: Direct prediction of CMax viabilities, related to Figure 5. Sub-figures A and B show the distribution of MAE and PCC per drug for the prediction of CMax viabilities using the cell line-drug combinations from the test set monotherapy data. Data are represented as boxplots where the box denotes the interquartile range between the first quartile (25th percentile) and third quartile (75th percentile) of the data. The black horizontal line inside each box denotes the median, and the whiskers extend to the largest/smallest values within 1.5 times the interquartile range.

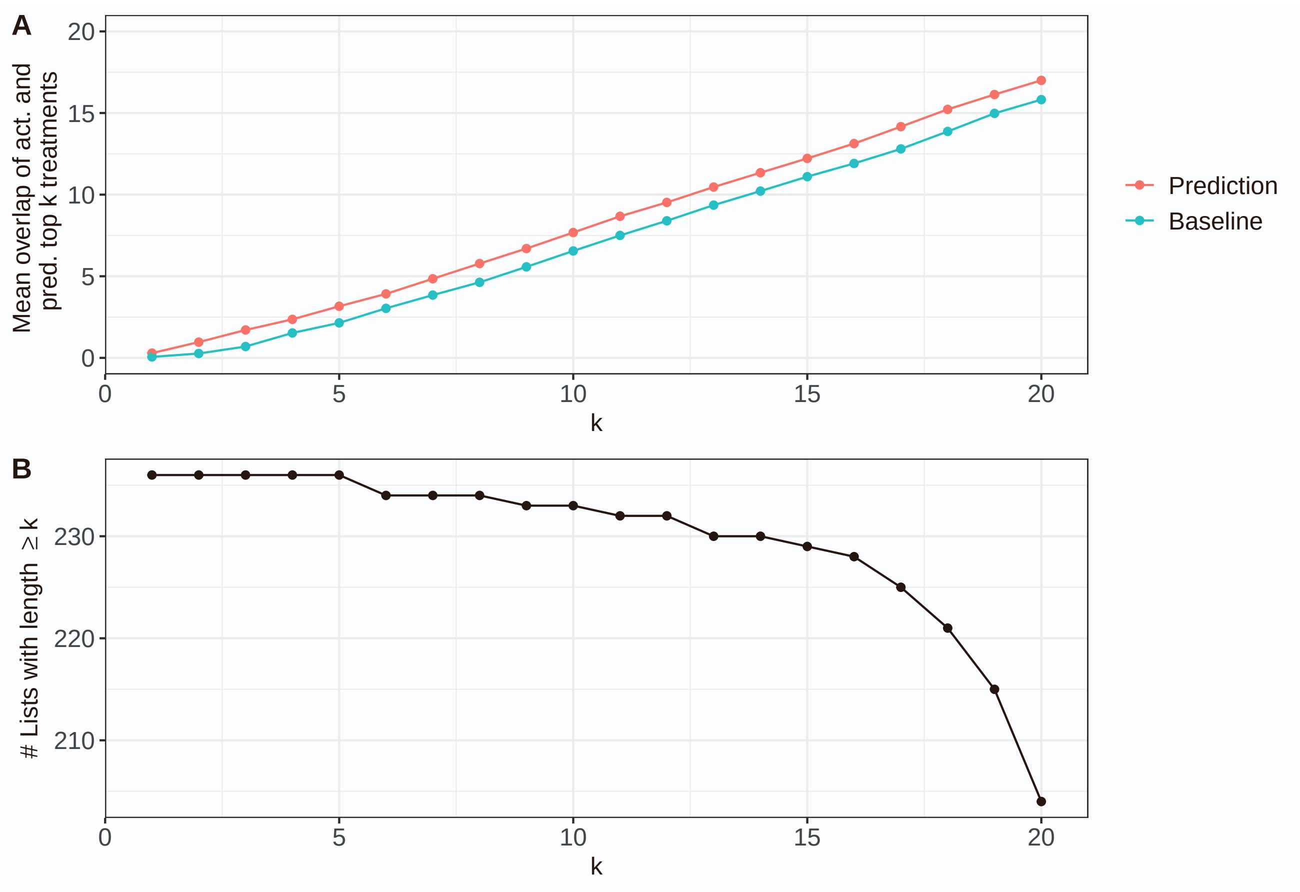

Figure S23: Overlap of k actual and predicted best treatments for monotherapies, related to Figure 6. Sub-figure A shows the average intersection size between the k actual best treatments and the k predicted best treatments for each cell line. Data are represented as mean over cell lines. Sub-figure B shows the number of cell lines based on which the average for each k was computed.

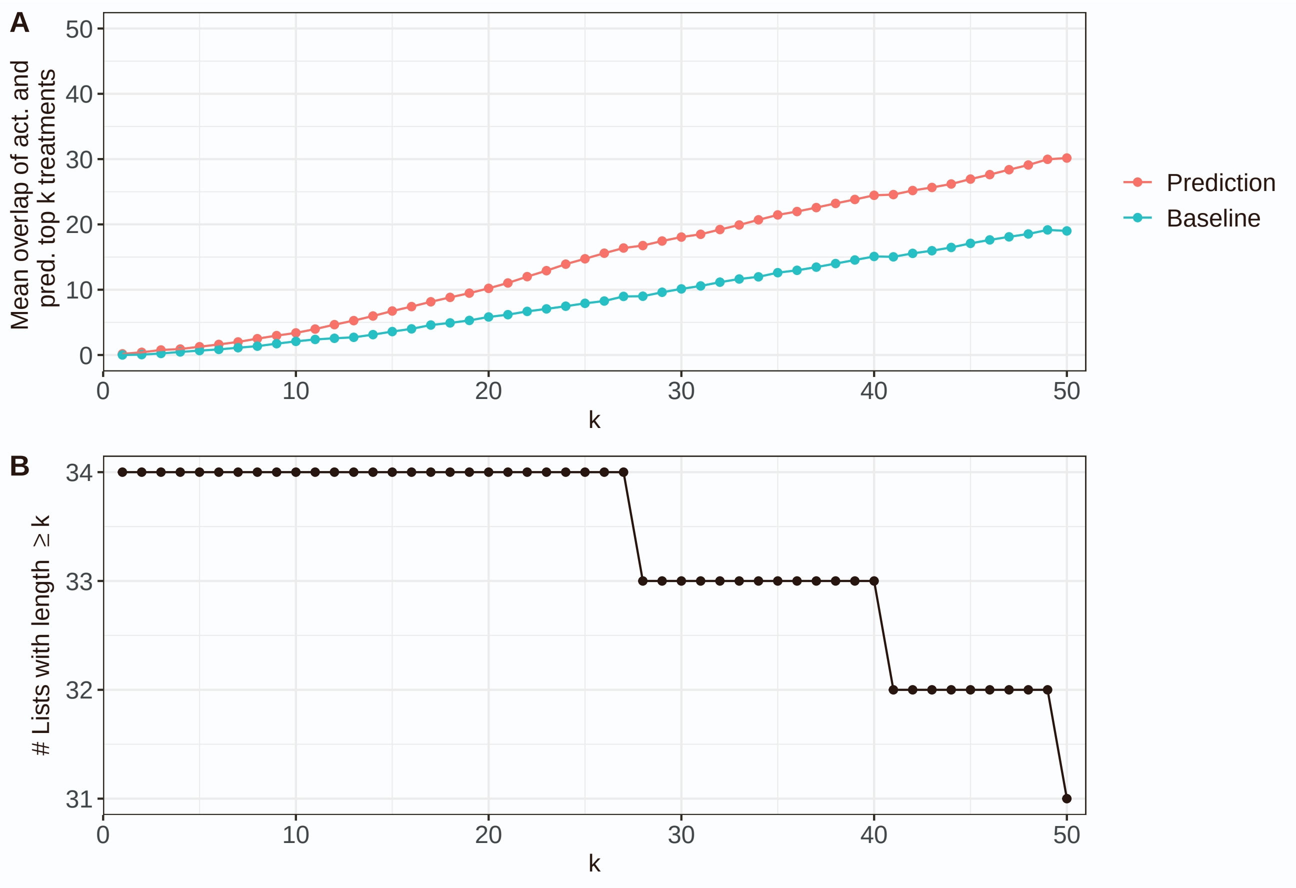

Figure S24: Overlap of k actual and predicted best treatments for the combination of both mono- and combination therapies, related to Figure 6. Sub-figure A shows the average intersection size between the k actual best treatments and the k predicted best treatments for each cell line. Data are represented as mean over cell lines. Sub-figure B shows the number of cell lines based on which the average for each k was computed. Note that the number of test cell lines with available combination data is smaller than the number of cell lines with available monotherapy data and we show the results only for cell lines where both were available.

Data S1: Additional information regarding the definition of relative inhibition values, related to STAR Methods.

Typically, cell viability assays measure the presence of live cells through fluorescence/luminescence intensities [59, 60, 61]. These intensities are further processed to obtain relative inhibitions. To this end, a background correction is performed by subtracting the intensity of the positive control wells (i.e., wells with only medium but no cells) from the intensity of the negative control wells (i.e., wells with untreated cells and medium) and all treatment wells (i.e., wells with treated cells and medium) [62].
Next, relative viabilities are computed by dividing the background-corrected intensities of all treatment wells with the negative control [62]. To obtain relative inhibitions, we subtract the relative viability from 1. In the DrugComb database, the resulting values are additionally multiplied by 100.
Typically, relative inhibitions are in range $(-\infty, 100]$, where values < 0 indicate that the treatment increases cell growth and values > 0 indicate a reduction in growth. While values > 100 should generally not occur, it can happen that the background correction becomes negative if the intensity of the treatment well is smaller than that of the control in cases where the treatment killed all cells. Note that Zheng et al., i.e., the authors of DrugComb, recommend considering only relative inhibitions in $[-200, 200]$ as data points outside of this range are deemed to be of poor quality [41]. In contrast, the Genomics of Drug Sensitivity in Cancer database limits the considered intervals more strictly [57].

Methods S1: Definitions of four commonly used synergy scores, related to STAR Methods.

In this section, we give a brief overview on four commonly used synergy scores, namely the HSA [63], Bliss [64], Loewe [65], and ZIP [66] synergy score. Our descriptions are limited to experiments using two-drug combinations. Extensions for an arbitrary number of drugs are provided at [67].

Consider an experiment where a cell line c is treated with different concentrations of two drugs, d_1 and d_2. For d_1, n different concentrations were tested, for d_2, m different concentrations were tested. The results of the combination treatments are reported in an $n \times m$ dose-response matrix Y, where each entry $y_{a,b}$ denotes the percentage of relative inhibition after administering dose a of d_1 in combination with dose b of d_2. Additionally, monotherapy responses for the same n (m) concentrations are required. We denote the percentage of relative inhibition obtained from the monotherapy of d_1 with concentration a as y_a. Analogously, the percentage of relative inhibition obtained from the monotherapy of d_2 with concentration b is given by y_b.
To measure the synergy between d_1 and d_2 on cell line c, the observed inhibitions $y_{a,b}$ are compared to the estimated inhibitions $\hat{y}_{a,b}$ that are calculated using a reference model which assumes no synergistic or antagonistic interaction between the two drugs. For the DrugComb database, synergy scores were computed using the widely applied synergyfinder R package [68], which calculates the synergy score (SS) for a cell-drug-drug combination as the average over all concentration-specific comparisons between the observed and the estimated drug responses [67]:

$$SS = \frac{1}{n \cdot m} \sum_{a \in \mathcal{A}} \sum_{b \in \mathcal{B}} (y_{a,b} - \hat{y}_{a,b}) \tag{1}$$

Here, $\mathcal{A}$ and $\mathcal{B}$ denote the sets containing all tested doses for drug d_1 and d_2, respectively. For $SS > 0$, the observed inhibitions are on average greater than the expected inhibitions, indicating synergy between d_1 and d_2. In contrast, for $SS < 0$, the observed inhibitions are smaller than expected, indicating an antagonistic interaction between the drugs.
Note that the synergyfinder documentation states that the percentage relative inhibitions utilized to derive SS are typically expected to be in range $[0, 1]$, while values outside of this range are still allowed [69]. While there is a (non-default) option to baseline-correct negative inhibitions [69], we did not find documentation on how synergyfinder handles negative values or values greater than 1 when present.

In the following, we present four of the most common reference models for estimating $\hat{y}_{a,b}$.

HSA [63]: The highest single agent (HSA) model expects the effect of a non-interacting drug combination to be equal to the greater of both monotherapy effects at the same concentrations [63]:

$$\hat{y}_{a,b}^{HSA} = max(y_a, y_b) \tag{2}$$
$$\tag{3}$$

Bliss [64]: The Bliss model assumes the combination effect of two drugs to be statistically independent [64]:

$$\hat{y}_{a,b}^{Bliss} = y_a + y_b - y_a \cdot y_b \tag{4}$$

Loewe [65]: The Loewe model assumes that there exists a concentration A of d_1 and a concentration B of d_2, for which the monotherapy can achieve the same effect as the combination of both drugs:

$$y_{a,b} = y_A = y_B \tag{5}$$

Additionally, it is assumed that for each concentration of drug d_1, there exists a concentration of drug d_2 with the same effect and vice versa. Consequently, we can define

$$A = a + a_b \tag{6}$$
$$B = b + b_a \quad , \tag{7}$$

where, a_b denotes the concentration of d_1 for which $y_{a_b} = y_b$. Analogously, b_a denotes the concentration of d_2 for which $y_{b_a} = y_a$.
The model furthermore assumes that the potency ratio between the two drugs is constant over the entire dose-response curve. This means the ratio between two concentrations from d_1 and d_2 achieving the same effect is a constant R. In particular, it holds that

$$R = \frac{A}{B} = \frac{a_b}{b} = \frac{a}{b_a} \quad . \tag{8}$$

It follows that the expected combination response exhibits so-called *Loewe additivity* [66]:

$$a + a_b = A \tag{9}$$
$$\Leftrightarrow a + b \cdot R = A \tag{10}$$
$$\Leftrightarrow a + b \cdot \frac{A}{B} = A \tag{11}$$
$$\Leftrightarrow \frac{a}{A} + \frac{b}{B} = 1 \tag{12}$$

To identify A and B, and, consequently, to compute $\hat{y}_{a,b}^{Loewe}$, dose-response curves for both drugs need to be fit, which are generally modeled as four-parametric logistic functions [66]. We omit the details here but refer interested readers to Yadav et al. for a detailed description [66], which concludes that $\hat{y}_{a,b}^{Loewe}$ can finally be computed by solving:

$$\frac{a}{m_1 \cdot \left(\frac{\hat{y}_{a,b}^{Loewe} - E_{min}^1}{E_{max}^1 - \hat{y}_{a,b}^{Loewe}}\right)^{\frac{1}{\lambda_1}}} + \frac{b}{m_2 \cdot \left(\frac{\hat{y}_{a,b}^{Loewe} - E_{min}^2}{E_{max}^1 - \hat{y}_{a,b}^{Loewe}}\right)^{\frac{1}{\lambda_2}}} = 1 \tag{13}$$

Here, E_{min}^1 and E_{max}^1 are derived from the fitted dose-response curve and denote the minimum and maximum effect that drug d_1 can achieve. Additionally, λ_1 denotes the slope and m_1 the midpoint of the dose-response curve for d_1. E_{min}^2, E_{max}^2, λ_2 and m_2 are defined analogously for drug d_2.

ZIP [66]: The *zero interaction potency* (ZIP) model combines ideas of both the Bliss and Loewe models. It models the notion of non-interaction between drugs by assuming that the dose-response curve of one drug is

unaffected by the addition of the second drug. Consequently, the combination effect can be described by shifting the dose-response curve of either drug by the effect of the other. Shifting the curve of drug d_1 by the effect of drug d_2 at concentration b can be described as follows (assuming that $E^1_{min} = 0$ and $E^1_{max} = 1$) [66]:

$$\hat{y}_{1\leftarrow 2} = \frac{y_b + \frac{x}{m_1}{}^{\lambda}_{1}}{1 + (\frac{x}{m_1})^{\lambda}_{1}} \tag{14}$$

Here, x denotes any dose of d_1. Thus, the conventional dose-response curve for d_1 is modified by simply adding y_b in the numerator, thereby increasing the baseline effect. Analogously, $\hat{y}_{2\leftarrow 1}$ can be defined. Consequently, just like the Loewe score, the ZIP score relies on accurate curve fittings for both monotherapies. Yadav et al. show that both $\hat{y}_{1\leftarrow 2}$ and $\hat{y}_{2\leftarrow 1}$ are equivalent to estimating the combined drug response as follows [66]:

$$\hat{y}^{ZIP}_{a,b} = \frac{\left(\frac{a}{m_1}\right)^{\lambda_1}}{1 + \left(\frac{a}{m_1}\right)^{\lambda_1}} + \frac{\left(\frac{b}{m_2}\right)^{\lambda_2}}{1 + \left(\frac{b}{m_2}\right)^{\lambda_2}} - \frac{\left(\frac{a}{m_1}\right)^{\lambda_1}}{1 + \left(\frac{a}{m_1}\right)^{\lambda_1}} \cdot \frac{\left(\frac{b}{m_2}\right)^{\lambda_2}}{1 + \left(\frac{b}{m_2}\right)^{\lambda_2}} \tag{15}$$

Similar to the Bliss score provided in Equation 4, Equation 15 also follows the form $X + Y - X \cdot Y$. Consequently, the notion of *non-interaction* between drugs in the ZIP model is also modeled through probabilistic independence [66].

Table S2: Investigated Compounds, related to STAR Methods. Table continues on next pages.

	Compound Name / CAS Number		Compound Name / CAS Number
1	Abiraterone	51	CHIR-99021
2	actinomycin D	52	chlorambucil
3	ADM hydrochloride	53	cis-Platin
4	Afatinib	54	Co-V
5	Akt inhibitor VIII	55	Crizotinib
6	allopurinol	56	cyclophosphamide
7	alpelisib	57	Cylocide
8	altretamine	58	CYTARABINE HYDROCHLORIDE
9	amifostine	59	dacarbazine
10	anastrozole	60	Daporinad
11	Antibiotic AD 32	61	Darinaparsin
12	Antibiotic AY 22989	62	Dasatinib
13	Avagacestat	63	Decitabine
14	Axitinib	64	Deforolimus
15	Azacytidine, 5-	65	dexamethasone
16	AZD1208	66	Dexrazoxane
17	AZD1480	67	Dinaciclib
18	AZD2014	68	docetaxel
19	AZD4320	69	Doramapimod
20	AZD4547	70	dorsomorphin
21	AZD5363	71	Dovitinib
22	AZD5582	72	doxorubicin
23	AZD6482	73	Elesclomol
24	AZD6738	74	Eloxatin (TN)
25	AZD7762	75	Eloxatin (TN) (Sanofi Synthelab)
26	AZD8055	76	EMBELIN
27	AZD8186	77	Emcyt (Pharmacia)
28	Belinostat	78	Entinostat
29	Bendamustine hydrochloride	79	Enzastaurin
30	Bexarotene	80	Erlotinib
31	BI-78D3	81	Erlotinib hydrochloride
32	BI-D1870	82	etoposide
33	BI 2536	83	EXEMESTANE
34	bicalutamide	84	Fedratinib
35	Bleo	85	FH535
36	bleomycin	86	Fingolimod
37	BMS-536924	87	Fludarabine Base
38	BMS-754807	88	Foretinib
39	Bortezomib	89	Fulvestrant
40	Bosutinib	90	GDC-0879
41	busulfan	91	Gefitinib
42	CABAZITAXEL	92	geldanamycin
43	Cabozantinib	93	gemcitabine
44	Carboplatinum	94	GSK 650394
45	carmustine	95	GSK429286A
46	Cediranib	96	GW 441756
47	celecoxib	97	GW0742
48	CHEMBL17639	98	GW2580
49	CHEMBL277800	99	GW843682X
50	CHEMBL3103192	100	hydroxyurea

Continuation of Table S2

	Compound Name / CAS Number		Compound Name / CAS Number
101	Idelalisib	151	OSI-027
102	ifosfamide	152	OSI-930
103	Imatinib	153	OSU-03012
104	IMD-0354	154	paclitaxel
105	IMIQUIMOD	155	Palbociclib
106	IPA-3	156	Panobinostat
107	Ixabepilone	157	parthenolide
108	JZL184	158	Pazopanib
109	Ku-0063794	159	Pazopanib hydrochloride
110	KU-55933	160	Pemetrexed
111	KU-60019	161	Perifosine
112	l-685,458	162	PF-04217903
113	L-778123 free base	163	PF-562271
114	Lapatinib	164	PHA-793887
115	Lenalidomide	165	PI-103
116	Lestaurtinib	166	PIK-93
117	letrozole	167	Pioglitazone
118	lfm-a13	168	Piperlongumine
119	Linifanib	169	pipobroman
120	Linsitinib	170	PLX-4720
121	lomustine	171	Pralatrexate
122	Masitinib	172	Procarbazine hydrochloride
123	MEGESTROL ACETATE	173	QS11
124	Melphalan hydrochloride	174	Quinacrine hydrochloride
125	metformin	175	Quizartinib
126	methotrexate	176	RAF265
127	methoxsalen	177	raloxifene
128	Midostaurin	178	Retinoic acid
129	MITHRAMYCIN	179	Romidepsin
130	mitomycin C	180	Ruxolitinib
131	mitotane	181	Sapitinib
132	mitoxantrone	182	Saracatinib
133	MK-1775	183	Selumetinib
134	MK-2206	184	Serdemetan
135	MK-4541	185	Silmitasertib
136	MK-5108	186	SNS-032
137	MLN4924	187	SNX-2112
138	MRK003	188	Sorafenib
139	Navelbine ditartrate (TN)	189	Sunitinib
140	Navitoclax	190	T0901317
141	Nilotinib	191	Tamoxan
142	Niraparib	192	Tamoxifen citrate
143	NSC-127716	193	Tanespimycin
144	NSC256439	194	temozolomide
145	NSC609699	195	Temsirolimus
146	NSC733504	196	teniposide
147	NSC756645	197	TGX-221
148	Nutlin-3	198	thalidomide
149	Olaparib	199	thapsigargin
150	Onalespib	200	thiotepa

Continuation of Table S2

	Compound Name / CAS Number
201	Tipifarnib
202	Tivozanib
203	topotecan
204	TOPOTECAN HYDROCHLORIDE
205	Tozasertib
206	TPCA-1
207	Trametinib
208	Triethylenemelamine
209	Trisenox
210	Tubastatin A
211	TW-37
212	UNC0638
213	Uramustine
214	US9505780, JQ-1
215	Vandetanib
216	Veliparib
217	Vemurafenib
218	Vepesid J
219	vinblastine
220	Vinblastine sulfate
221	vincristine
222	Vincristine sulfate
223	vinorelbine
224	Vismodegib
225	Vorinostat
226	VX-702
227	XL147
228	XL765
229	YK 4-279
230	Zanosar
231	Zoledronic acid
232	ZSTK474
233	(-)-Rapamycin
234	001, RAD
235	1032350-13-2
236	122111-05-1
237	1260907-17-2
238	158798-73-3
239	218137-86-1
240	219580-11-7
241	23541-50-6
242	284028-89-3
243	303727-31-3
244	315183-21-2
245	391210-10-9
246	49843-98-3
247	5-Aminolevulinic acid hydrochloride
248	5-azacytidine
249	5-Fluoro-2'-deoxyuridine
250	5-Fluorouracil

	Compound Name / CAS Number
251	547757-23-3
252	55-86-7
253	6-Mercaptopurine
254	6-Thioguanine
255	7-Ethyl-10-hydroxycamptothecin
256	717906-29-1
257	761439-42-3
258	7803-88-5
259	781661-94-7
260	803712-79-0
261	841290-80-0
262	844499-71-4
263	891494-63-6
264	915019-65-7
265	957054-30-7

Table S3: Hyperparameters of the investigated ML algorithms, related to STAR Methods. This table denotes the tuned hyperparameters for each ML algorithm. For hyperparameters not stated explicitly, the default parameters as provided the respective Python package were employed. For the PhysChem setting (i.e., the setting with the largest data matrix), we were unable to train neural networks with the ELU activation or learning rates of 0.1 due to insufficient memory for resource allocation even when decreasing the batch size.

Model	Parameter	Value(s)
Elastic net	alpha	0.01, 0.1, 1, 10, 100
	l1_ratio	0, 0.25, 0.5, 0.75, 1
Random forest	max_depth	100, 1000000
	max_features	25, 50, 100, 250
	min_samples_leaf	2, 20, 100, 1000
	n_estimators	500
Neural network	loss	mean_squared_error
	activation	tanh, ELU (none in last layer)
	optimizer	Adam
	learning_rate	0.0001, 0.001, 0.1
	hidden_layers	1,2,3,4,5
	size of hidden layers	equally spaced btw. in-/output size
	dropout	0.1, 0.3
	batch_size	256
	bias_initializer	0.01
	kernel_initializer	glorot_uniform for tanh,<br>he_normal for ELU activation
	kernel_regularizer	l2
	epochs	300
	validation_split	0.2
	early stopping	yes
	patience	15
	restore_best_weights	True

Table S4: Tuned hyperparameters for each ML algorithm and setting, related to STAR Methods. For hyperparameters not stated explicitly, the values denoted in Table S3 were employed. Otherwise, we used the default parameters as provided the respective Python packages.

Model	Setting	Parameters
Random Forest	OneHot	max_features=250; max_depth=1000000; min_samples_leaf=2
Random Forest	OneHotTar	max_features=250; max_depth=1000000; min_samples_leaf=2
Random Forest	MACCS	max_features=250; max_depth=100; min_samples_leaf=2
Random Forest	PhysChem	max_features=100; max_depth=100; min_samples_leaf=2
Neural Network	OneHot	activation=elu; learning_rate=0.0001; num_hidden_layers=5; dropout=0.3;
Neural Network	OneHotTar	activation=elu; learning_rate=0.0001; num_hidden_layers=4; dropout=0.3;
Neural Network	MACCS	activation=elu; learning_rate=0.0001; num_hidden_layers=5; dropout=0.3;
Neural Network	PhysChem	activation=tanh; learning_rate=0.0001; num_hidden_layers=4; dropout=0.1;
Elastic Net	OneHot	alpha=0.01; l1_ratio=1
Elastic Net	OneHotTar	alpha=0.01; l1_ratio=1
Elastic Net	MACCS	alpha=0.01; l1_ratio=1
Elastic Net	PhysChem	alpha=0.01; l1_ratio=1

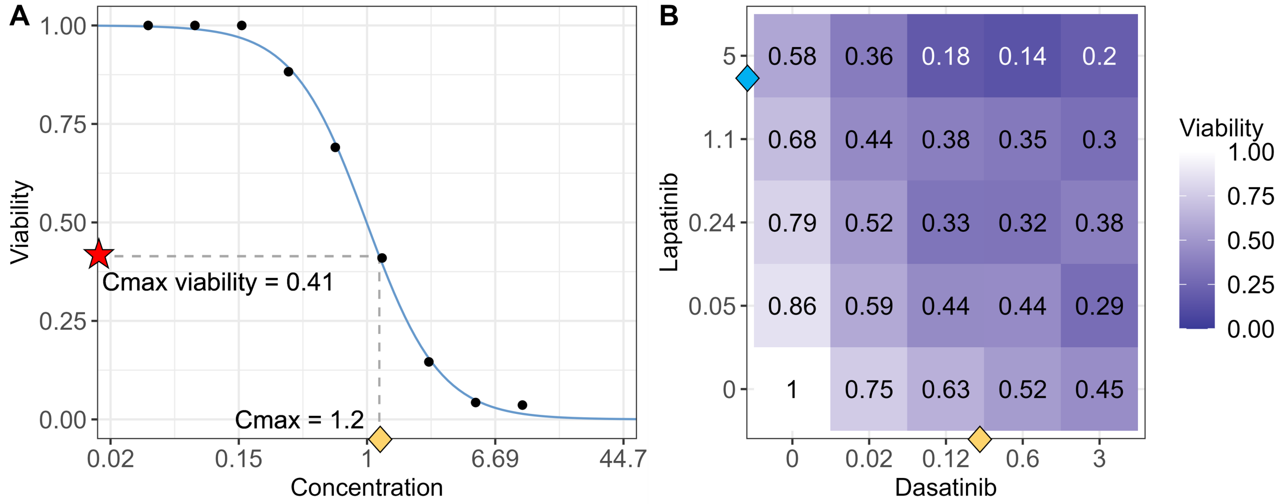

Figure S25: Exemplary dose-response curve and matrix, related to STAR Methods. Sub-figure A depicts a dose-response curve (blue) for the monotherapy treatment of a cancer cell line (COSMIC ID 683667) with the drug Vorinostat. The fit is based on nine dose-response points (black). The yellow diamond marks the CMax concentration of Vorinostat (1.2μM) , and the red star marks the corresponding CMax viability (0.41) derived from the curve. Sub-figure B depicts a dose-response matrix for the combination treatment of cell line 909755 with Dasatinib and Lapatinib, where the x- and y-axes denote the respective treatment concentrations. The yellow and blue diamonds approximately mark the CMax concentration of both drugs, which are used to limit the considered concentration combinations for the computation of the combination CMax viability.

References

[1] Michael P Menden, Francesco Iorio, Mathew Garnett, Ultan McDermott, Cyril H Benes, Pedro J Ballester, and Julio Saez-Rodriguez. Machine learning prediction of cancer cell sensitivity to drugs based on genomic and chemical properties. *PLoS one*, 8(4):e61318, 2013.

[2] Tiziana Pivetta, Francesco Isaia, Federica Trudu, Alessandra Pani, Matteo Manca, Daniela Perra, Filippo Amato, and Josef Havel. Development and validation of a general approach to predict and quantify the synergism of anti-cancer drugs using experimental design and artificial neural networks. *Talanta*, 115:84–93, 2013.

[3] Naiqian Zhang, Haiyun Wang, Yun Fang, Jun Wang, Xiaoqi Zheng, and X Shirley Liu. Predicting anticancer drug responses using a dual-layer integrated cell line-drug network model. *PLoS computational biology*, 11(9):e1004498, 2015.

[4] Jiangyong Gu, Xinzhuang Zhang, Yimin Ma, Na Li, Fang Luo, Liang Cao, Zhenzhong Wang, Gu Yuan, Lirong Chen, Wei Xiao, et al. Quantitative modeling of dose–response and drug combination based on pathway network. *Journal of cheminformatics*, 7:1–10, 2015.

[5] Jichen Yang, H Tang, Y Li, R Zhong, T Wang, STC Wong, G Xiao, and Y Xie. Digre: Drug-induced genomic residual effect model for successful prediction of multidrug effects. *CPT: pharmacometrics & systems pharmacology*, 4(2):91–97, 2015.

[6] R. Rahman and R. Pal. Analyzing drug sensitivity prediction based on dose response curve characteristics. In *IEEE-EMBS International Conference on Biomedical and Health Informatics (BHI)*, pages 140–143, 2016.

[7] Anat Zimmer, Itay Katzir, Erez Dekel, Avraham E Mayo, and Uri Alon. Prediction of multidimensional drug dose responses based on measurements of drug pairs. *Proceedings of the National Academy of Sciences*, 113(37):10442–10447, 2016.

[8] Theo A Knijnenburg, Gunnar W Klau, Francesco Iorio, Mathew J Garnett, Ultan McDermott, Ilya Shmulevich, and Lodewyk FA Wessels. Logic models to predict continuous outputs based on binary inputs with an application to personalized cancer therapy. *Scientific reports*, 6(1):1–14, 2016.

[9] Yu-Ching Hsu, Yu-Chiao Chiu, Yidong Chen, Tzu-Hung Hsiao, and Eric Y Chuang. A simple gene set-based method accurately predicts the synergy of drug pairs. *BMC Systems Biology*, 10:313–322, 2016.

[10] L. Wang, X. Li, L. Zhang, and Q. Gao. Improved anticancer drug response prediction in cell lines using matrix factorization with similarity regularization. *BMC Cancer*, 17(1):1–12, 2017.

[11] Anat Zimmer, Avichai Tendler, Itay Katzir, Avi Mayo, and Uri Alon. Prediction of drug cocktail effects when the number of measurements is limited. *PLoS biology*, 15(10):e2002518, 2017.

[12] Xiangyi Li, Yingjie Xu, Hui Cui, Tao Huang, Disong Wang, Baofeng Lian, Wei Li, Guangrong Qin, Lanming Chen, and Lu Xie. Prediction of synergistic anti-cancer drug combinations based on drug target network and drug induced gene expression profiles. *Artificial intelligence in medicine*, 83:35–43, 2017.

[13] Zachary Stanfield, Mustafa Coşkun, and Mehmet Koyutürk. Drug response prediction as a link prediction problem. *Scientific reports*, 7(1):40321, 2017.

[14] Raziur Rahman, Kevin Matlock, Souparno Ghosh, and Ranadip Pal. Heterogeneity aware random forest for drug sensitivity prediction. *Scientific reports*, 7(1):1–11, 2017.

[15] Minji Jeon, Sunkyu Kim, Sungjoon Park, Heewon Lee, and Jaewoo Kang. In silico drug combination discovery for personalized cancer therapy. *BMC systems biology*, 12:59–67, 2018.

[16] Joseph D Janizek, Safiye Celik, and Su-In Lee. Explainable machine learning prediction of synergistic drug combinations for precision cancer medicine. *BioRxiv*, page 331769, 2018.

[17] Amrita Basu, Ritwik Mitra, Han Liu, Stuart L Schreiber, and Paul A Clemons. Rwen: response-weighted elastic net for prediction of chemosensitivity of cancer cell lines. *Bioinformatics*, 34(19):3332–3339, 2018.

[18] Yoosup Chang, Hyejin Park, Hyun-Jin Yang, Seungju Lee, Kwee-Yum Lee, Tae Soon Kim, Jongsun Jung, and Jae-Min Shin. Cancer drug response profile scan (cdrscan): a deep learning model that predicts drug effectiveness from cancer genomic signature. *Scientific reports*, 8(1):8857, 2018.

[19] Yun Fang, Peirong Xu, Jialiang Yang, and Yufang Qin. A quantile regression forest based method to predict drug response and assess prediction reliability. *PLoS One*, 13(10):e0205155, 2018.

[20] Hui Liu, Yan Zhao, Lin Zhang, and Xing Chen. Anti-cancer drug response prediction using neighbor-based collaborative filtering with global effect removal. *Molecular Therapy-Nucleic Acids*, 13:303–311, 2018.

[21] Hongyang Li, Shuai Hu, Nouri Neamati, and Yuanfang Guan. Taiji: approaching experimental replicates-level accuracy for drug synergy prediction. *Bioinformatics*, 35(13):2338–2339, 2019.

[22] Masturah Bte Mohd Abdul Rashid, Tan Boon Toh, Lissa Hooi, Aleidy Silva, Yanzhou Zhang, Pei Fang Tan, Ai Ling Teh, Neerja Karnani, Sudhakar Jha, Chih-Ming Ho, et al. Optimizing drug combinations against multiple myeloma using a quadratic phenotypic optimization platform (qpop). *Science translational medicine*, 10(453):eaan0941, 2018.

[23] Fangfang Xia, Maulik Shukla, Thomas Brettin, Cristina Garcia-Cardona, Judith Cohn, Jonathan E Allen, Sergei Maslov, Susan L Holbeck, James H Doroshow, Yvonne A Evrard, et al. Predicting tumor cell line response to drug pairs with deep learning. *BMC bioinformatics*, 19:71–79, 2018.

[24] Fei Zhang, Minghui Wang, Jianing Xi, Jianghong Yang, and Ao Li. A novel heterogeneous network-based method for drug response prediction in cancer cell lines. *Scientific reports*, 8(1):3355, 2018.

[25] Ran Su, Xinyi Liu, Leyi Wei, and Quan Zou. Deep-resp-forest: a deep forest model to predict anti-cancer drug response. *Methods*, 166:91–102, 2019.

[26] Hossein Sharifi-Noghabi, Olga Zolotareva, Colin C Collins, and Martin Ester. Moli: multi-omics late integration with deep neural networks for drug response prediction. *Bioinformatics*, 35(14):i501–i509, 2019.

[27] Yu-Chiao Chiu, Hung-I Harry Chen, Tinghe Zhang, Songyao Zhang, Aparna Gorthi, Li-Ju Wang, Yufei Huang, and Yidong Chen. Predicting drug response of tumors from integrated genomic profiles by deep neural networks. *BMC medical genomics*, 12(1):143–155, 2019.

[28] Ali Oskooei, Matteo Manica, Roland Mathis, and María Rodríguez Martínez. Network-based biased tree ensembles (netbite) for drug sensitivity prediction and drug sensitivity biomarker identification in cancer. *Scientific reports*, 9(1):15918, 2019.

[29] Raziur Rahman, Saugato Rahman Dhruba, Souparno Ghosh, and Ranadip Pal. Functional random forest with applications in dose-response predictions. *Scientific reports*, 9(1):1628, 2019.

[30] Pavel Sidorov, Stefan Naulaerts, Jérémy Ariey-Bonnet, Eddy Pasquier, and Pedro J Ballester. Predicting synergism of cancer drug combinations using nci-almanac data. *Frontiers in chemistry*, 7:509, 2019.

[31] Aleksandr Ianevski, Anil K Giri, Prson Gautam, Alexander Kononov, Swapnil Potdar, Jani Saarela, Krister Wennerberg, and Tero Aittokallio. Prediction of drug combination effects with a minimal set of experiments. *Nature machine intelligence*, 1(12):568–577, 2019.

[32] Lei Deng, Yideng Cai, Wenhao Zhang, Wenyi Yang, Bo Gao, and Hui Liu. Pathway-guided deep neural network toward interpretable and predictive modeling of drug sensitivity. *Journal of Chemical Information and Modeling*, 60(10):4497–4505, 2020.

[33] Khandakar Tanvir Ahmed, Sunho Park, Qibing Jiang, Yunku Yeu, TaeHyun Hwang, and Wei Zhang. Network-based drug sensitivity prediction. *BMC medical genomics*, 13(11):1–10, 2020.

[34] Alexander Ling and R Stephanie Huang. Computationally predicting clinical drug combination efficacy with cancer cell line screens and independent drug action. *Nature communications*, 11(1):5848, 2020.

[35] Fatemeh Ahmadi Moughari and Changiz Eslahchi. Adrml: anticancer drug response prediction using manifold learning. *Scientific reports*, 10(1):14245, 2020.

[36] Heli Julkunen, Anna Cichonska, Prson Gautam, Sandor Szedmak, Jane Douat, Tapio Pahikkala, Tero Aittokallio, and Juho Rousu. Leveraging multi-way interactions for systematic prediction of pre-clinical drug combination effects. *Nature communications*, 11(1):6136, 2020.

[37] Fatemeh Yassaee Meybodi and Changiz Eslahchi. Predicting anti-cancer drug response by finding optimal subset of drugs. *Bioinformatics*, 37(23):4509–4516, 2021.

[38] Y.-C. Tang and A. Gottlieb. Explainable drug sensitivity prediction through cancer pathway enrichment. *Scientific reports*, 11(1):1–10, 2021.

[39] Tuan Nguyen, Giang TT Nguyen, Thin Nguyen, and Duc-Hau Le. Graph convolutional networks for drug response prediction. *IEEE/ACM transactions on computational biology and bioinformatics*, 19(1):146–154, 2021.

[40] Omid Bazgir, Souparno Ghosh, and Ranadip Pal. Investigation of REFINED CNN ensemble learning for anti-cancer drug sensitivity prediction. *Bioinformatics*, 37(Supplement 1):i42–i50, 07 2021.

[41] Shuyu Zheng, Jehad Aldahdooh, Tolou Shadbahr, Yinyin Wang, Dalal Aldahdooh, Jie Bao, Wenyu Wang, and Jing Tang. Drugcomb update: a more comprehensive drug sensitivity data repository and analysis portal. *Nucleic acids research*, 49(W1):W174–W184, 2021.

[42] Cristiana Correia, Abigail Ferreira, Joana Santos, Rui Lapa, Marjo Yliperttula, Arto Urtti, and Nuno Vale. New in vitro-in silico approach for the prediction of in vivo performance of drug combinations. *Molecules*, 26(14):4257, 2021.

[43] Kerstin Lenhof, Nico Gerstner, Tim Kehl, Lea Eckhart, Lara Schneider, and Hans-Peter Lenhof. Merida: a novel boolean logic-based integer linear program for personalized cancer therapy. *Bioinformatics*, 37(21):3881–3888, 2021.

[44] Kanggeun Lee, Dongbin Cho, Jinho Jang, Kang Choi, Hyoung-oh Jeong, Jiwon Seo, Won-Ki Jeong, and Semin Lee. Ramp: response-aware multi-task learning with contrastive regularization for cancer drug response prediction. *Briefings in Bioinformatics*, 24(1):bbac504, 2023.

[45] Xiaoxiao Cheng, Chong Dai, Yuqi Wen, Xiaoqi Wang, Xiaochen Bo, Song He, and Shaoliang Peng. Nerd: a multichannel neural network to predict cellular response of drugs by integrating multidimensional data. *BMC medicine*, 20(1):368, 2022.

[46] Smriti Chawla, Anja Rockstroh, Melanie Lehman, Ellca Ratther, Atishay Jain, Anuneet Anand, Apoorva Gupta, Namrata Bhattacharya, Sarita Poonia, Priyadarshini Rai, et al. Gene expression based inference of cancer drug sensitivity. *Nature communications*, 13(1):5680, 2022.

[47] Pietro Pinoli, Gaia Ceddia, Stefano Ceri, and Marco Masseroli. Predicting drug synergism by means of non-negative matrix tri-factorization. *IEEE/ACM Transactions on Computational Biology and Bioinformatics*, 19(4):1956–1967, 2021.

[48] Mehmet Gönen and Adam A Margolin. Drug susceptibility prediction against a panel of drugs using kernelized bayesian multitask learning. *Bioinformatics*, 30(17):i556–i563, 2014.

[49] Kristina Preuer, Richard PI Lewis, Sepp Hochreiter, Andreas Bender, Krishna C Bulusu, and Günter Klambauer. Deepsynergy: predicting anti-cancer drug synergy with deep learning. *Bioinformatics*, 34(9):1538–1546, 2018.

[50] Qiao Liu, Zhiqiang Hu, Rui Jiang, and Mu Zhou. Deepcdr: a hybrid graph convolutional network for predicting cancer drug response. *Bioinformatics*, 36(Supplement_2):i911–i918, 2020.

[51] Yejin Kim, Shuyu Zheng, Jing Tang, Wenjin Jim Zheng, Zhao Li, and Xiaoqian Jiang. Anticancer drug synergy prediction in understudied tissues using transfer learning. *Journal of the American Medical Informatics Association*, 28(1):42–51, 2021.

[52] Halil Ibrahim Kuru, Oznur Tastan, and A Ercument Cicek. Matchmaker: a deep learning framework for drug synergy prediction. *IEEE/ACM transactions on computational biology and bioinformatics*, 19(4):2334–2344, 2021.

[53] Kerstin Lenhof, Lea Eckhart, Nico Gerstner, Tim Kehl, and Hans-Peter Lenhof. Simultaneous regression and classification for drug sensitivity prediction using an advanced random forest method. *Scientific Reports*, 12(1):13458, 2022.

[54] Kerstin Lenhof, Lea Eckhart, Lisa-Marie Rolli, Andrea Volkamer, and Hans-Peter Lenhof. Reliable anticancer drug sensitivity prediction and prioritization. *Scientific Reports*, 14(1):12303, 2024.

[55] Hong Wang, Chong Dai, Yuqi Wen, Xiaoqi Wang, Wenjuan Liu, Song He, Xiaochen Bo, and Shaoliang Peng. Gadrp: graph convolutional networks and autoencoders for cancer drug response prediction. *Briefings in Bioinformatics*, 24(1):bbac501, 2023.

[56] Dane R Liston and Myrtle Davis. Clinically relevant concentrations of anticancer drugs: A guide for nonclinical studiesguide to clinical exposures of anticancer drugs. *Clinical cancer research*, 23(14):3489–3498, 2017.

[57] Daniel J Vis, Lorenzo Bombardelli, Howard Lightfoot, Francesco Iorio, Mathew J Garnett, and Lodewyk FA Wessels. Multilevel models improve precision and speed of ic50 estimates. *Pharmacogenomics*, 17(7):691–700, 2016.

[58] Wellcome Sanger Institute, GDSC database. Resources download - ic50 data definitions. `https://cog.sanger.ac.uk/cancerrxgene/GDSC_release8.5/GDSC_Fitted_Data_Description.pdf`, 2024. Accessed: 2025-03-20.

[59] Syto® red fluorescent nucleic acid stains. `https://www.thermofisher.com/document-connect/document-connect.html?url=https://assets.thermofisher.com/TFS-Assets%2FLSG%2Fmanuals%2Fmp11340.pdf`. Accessed: 2024-11-22.

[60] Shailendra Anoopkumar-Dukie, JB Carey, T Conere, E O'sullivan, FN Van Pelt, and A Allshire. Resazurin assay of radiation response in cultured cells. *The British journal of radiology*, 78(934):945–947, 2005.

[61] Celltiter-glo ® luminescent cell viability assay. `https://www.promega.de/products/cell-health-assays/cell-viability-and-cytotoxicity-assays/celltiter_glo-luminescent-cell-viability-assay/?catNum=G7570`. Accessed: 2025-03-20.

[62] Howard Lightfoot, Dieudonne van der Meer, and Daniel J. Vis. *gdscIC50: Pipeline for GDSC Curve Fitting*, 2021. R package version 0.99.4.

[63] Morris C Berenbaum. What is synergy? *Pharmacological reviews*, 41(2):93–141, 1989.

[64] Chester I Bliss. The toxicity of poisons applied jointly 1. *Annals of applied biology*, 26(3):585–615, 1939.

[65] S Loewe. The problem of synergism and antagonism of combined drugs. *Arzneimittel-forschung*, 3(6):285–290, 1953.

[66] Bhagwan Yadav, Krister Wennerberg, Tero Aittokallio, and Jing Tang. Searching for drug synergy in complex dose–response landscapes using an interaction potency model. *Computational and structural biotechnology journal*, 13:504–513, 2015.

[67] Synergyfinder - user documentation. `https://synergyfinder.fimm.fi/synergy/synfin_docs/`. Accessed: 2025-03-20.

[68] Shuyu Zheng, Wenyu Wang, Jehad Aldahdooh, Alina Malyutina, Tolou Shadbahr, Alberto Pessia, and Jing Tang. Synergyfinder plus: towards a better interpretation and annotation of drug combination screening datasets. *bioRxiv*, page 2021.06.01.446564, 2021.

[69] User tutorial of the synergyfinder plus. https://www.bioconductor.org/packages/release/bioc/vignettes/synergyfinder/inst/doc/User_tutorual_of_the_SynergyFinder_plus.html. Accessed: 2025-03-20.
